# Calcitriol alleviates ARID2-deficient gastric cancer metastasis via inhibition of the Wnt/β-catenin signaling pathway

**DOI:** 10.1016/j.tranon.2026.103048

**Published:** 2026-09-22

**Authors:** Biying Liu, Zhaole Chu, Yixian Fan, Ye Tian, Jing Jin, Tao Wang, Ximiao He

**Affiliations:** a Department of Physiology, School of Basic Medicine, Tongji Medical College, Huazhong University of Science and Technology, Wuhan, Hubei, 430030, China; b Department of Gastroenterology, Chongqing Key Laboratory of Digestive Malignancies, Daping Hospital, Army Medical University (Third Military Medical University), Chongqing, 400042, China; c Center for Genomics and Proteomics Research, School of Basic Medicine, Tongji Medical College, Huazhong University of Science and Technology, Wuhan, 430030, China; d State Key Laboratory of Resource Insects, Southwest University, Chongqing, 400715, China; e The Second Affiliated Hospital of Guangzhou Medical University, Guangdong Provincial Key Laboratory of Allergy and Clinical Immunology, Guangzhou, 510260, China; f National Demonstration Center for Experimental Basic Medical Education, School of Basic Medicine, Tongji Medical College, Huazhong University of Science and Technology, Wuhan, Hubei, 430030, China; g Hubei Key Laboratory of Drug Target Research and Pharmacodynamic Evaluation, Huazhong University of Science and Technology, Wuhan, Hubei, 430030, China; h Key Laboratory of Vascular Aging, Ministry of Education, Tongji Hospital, Tongji Medical College, Huazhong University of Science and Technology, Wuhan, Hubei, 430030, China

**Keywords:** Gastric cancer metastasis, ARID2, VDR, Wnt signaling, Calcitriol

## Abstract

**Introduction:**

Somatic mutations of the chromatin remodeling gene ARID2 occur at a higher frequency in gastric cancer (GC) and correlate with poor prognosis. However, the mechanisms by which ARID2 impacts GC progression remain largely unknown.

**Methods:**

Functional and mechanistic studies were conducted to investigate how ARID2 deficiency promotes GC metastasis via activation of the Wnt pathway. Integrative RNA-seq and CUT&Tag identified vitamin D receptor (VDR) as a key transcription factor in ARID2-knockdown GC cells. CUT&RUN qPCR and luciferase reporter assays were used to detect ARID2 occupancy at the *VDR* promoter and the effects of ARID2 on *VDR* promoter activity, respectively. The *in vivo* findings were further validated in peritoneal metastasis models.

**Results:**

Our study demonstrated that ARID2 deficiency was associated with shorter progression-free survival in GC patients with lymph node metastasis. Mechanistically, ARID2 was enriched at the *VDR* promoter and promoted *VDR* transcription. Consequently, ARID2 knockdown led to VDR downregulation, which abolished VDR-mediated suppression of the Wnt signaling, resulting in the nuclear accumulation of β-catenin and GC metastasis. Strikingly, restoration of VDR signaling via genetic overexpression or administration of the VDR agonist 1,25-dihydroxyvitamin D3 (1,25(OH)_2_D3, calcitriol) specifically blocked β-catenin nuclear localization, thereby profoundly suppressing the metastatic progression of ARID2-deficient GC *in vitro* and *in vivo*.

**Conclusions:**

This study identifies the VDR-mediated Wnt/β-catenin signaling axis as a crucial mechanism through which ARID2 knockdown promotes GC cell metastasis. More significantly, VDR activation by calcitriol effectively reverses oncogenic changes.

## Highlights

• ARID2 mutation promotes the metastatic capability of GC.
• Disruption of ARID2-VDR interaction activates Wnt/β-catenin signaling pathway.
• VDR activation prevents ARID2-deficient GC metastasis by blocking nuclear β-catenin.
• Calcitriol may represent a therapeutic candidate for ARID2-deficient GC metastasis.

## Graphical abstract

**Schematic model for the coordinated role of ARID2 and VDR in GC metastasis.** Disruption of the ARID2-VDR interaction activates Wnt/β-catenin signaling, thereby promoting GC metastasis. Conversely, VDR overexpression or pharmacological activation by calcitriol reverses this prometastatic cascade. These findings suggest that targeting VDR is a promising therapeutic strategy for ARID2-deficient GC.

**Figure:**
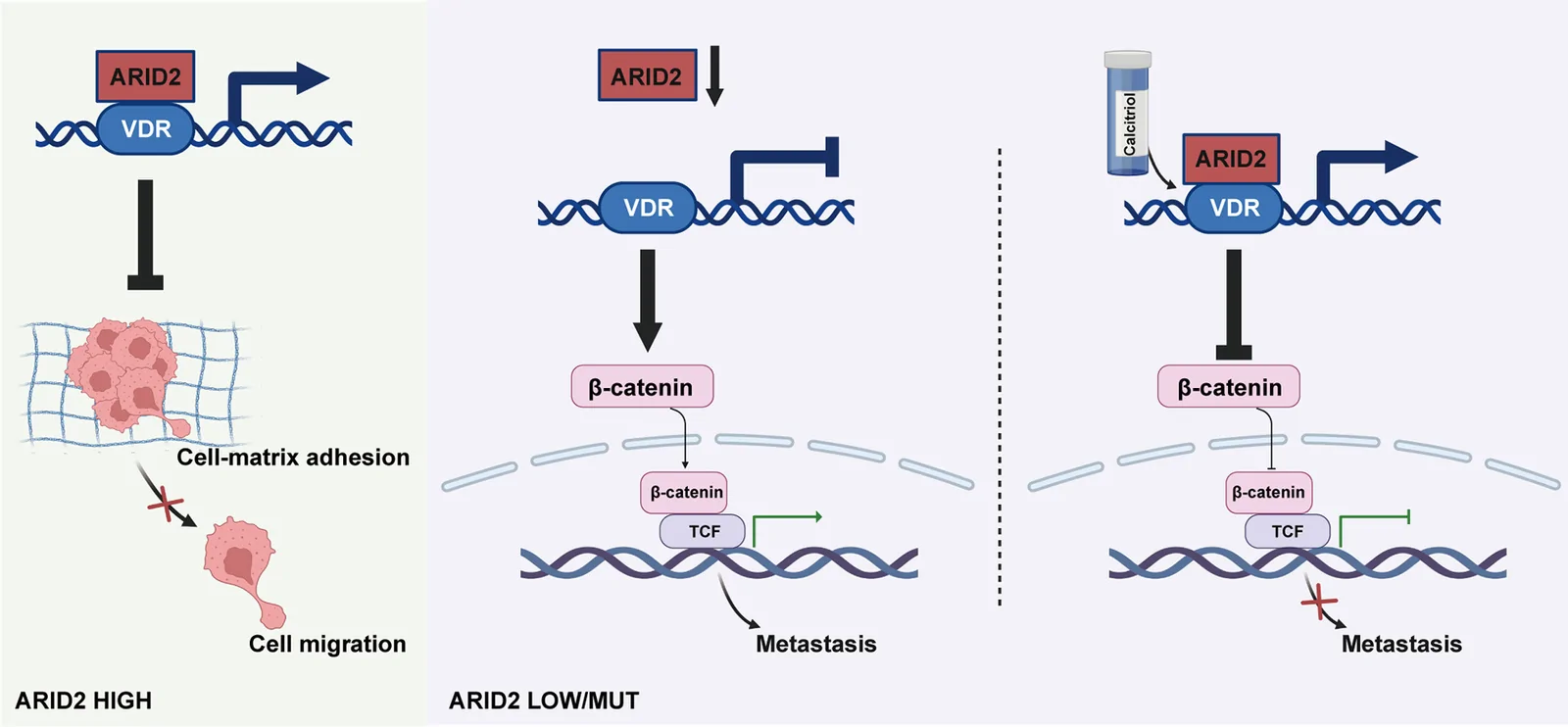

## Introduction

The SWItch/Sucrose Non-Fermenting (SWI/SNF) complex is a multisubunit chromatin remodeling complex that governs gene expression using the energy derived from ATP hydrolysis to alter DNA-nucleosome contacts and enable chromatin accessibility [1]. Approximately 25% of all human cancers harbor mutations in genes that encode subunits of the SWI/SNF complex, many of which are loss-of-function mutations, suggesting their broad tumor-suppressive roles [2]. Brg/Brahma-associated factors (cBAF) and Polybromo-associated BAF (PBAF) are major forms of SWI/SNF complexes, which are related to the specific functions of different polymorphic assemblies in different tissues. The cBAF complex is defined by the incorporation of unique subunits ARID1A/ARID1B/DPF2, whereas ARID2/PBRM1 are only found in PBAF [3]. PBAFs utilize distinct molecular mechanisms and play an essential role in specific developmental or oncogenic contexts.

Mutations in ARID2, which are predominantly inactivating and result in a loss of function, occur in a diverse array of solid tumor types [[4], [5], [6]]. ARID2 mutations are observed in ∼7.3% of lung cancers, and ARID2 expression decreases during malignant progression [7]. ARID2 loss promotes tumor progression in a TP53-mutated background, indicating a cooperative role between ARID2 and TP53 in oral squamous cell carcinoma [8]. Reduced ARID2 expression is associated with worse clinicopathological characteristics in patients with colorectal cancer [9]. Somatic mutations of *ARID2* are frequently found in esophageal squamous cell carcinoma (ESCC), and these genetic alterations occur at an early stage of ESCC development [10]. Crucially, clinical evidence has demonstrated that ARID2 functions as a tumor suppressor in gastric carcinogenesis, and ARID2 deficiency is significantly associated with gastric cancer (GC) progression [11]. Large-scale genomic sequencing has revealed that mutations in ARID2 undergo significant positive selection in gastric tissues, indicating that ARID2 acts as a crucial driver gene in gastric carcinogenesis [12]. Since metastasis remains the leading cause of mortality in GC patients and represents the most critical step in tumor progression [13], understanding its underlying epigenetic drivers is urgently needed. Despite the prevalence of genetic mutations in ARID2, the functional significance and underlying mechanisms of ARID2 in driving GC metastasis remain largely undefined.

Emerging evidence has shown that SWI/SNF complexes are involved in numerous biological processes, such as transcription, DNA replication, and DNA damage repair [14,15]. SWI/SNF complexes use the energy of ATP hydrolysis to remodel nucleosomes and bind to specific DNA regions, thereby modulating gene transcription through dynamic interactions with epigenetic modifications [16]. As a key subunit, ARID2 contains a large proline- and glutamine-rich domain, characteristic of many transcription factors (TFs), which may facilitate DNA binding or protein‒protein interactions [16]. Moreover, ARID2 interacts with the promoter of NEDD4L and enhances NEDD4L transcription, which inhibits Janus kinase 2 signaling, thereby reducing liver steatosis [17]. ARID2 loss disrupts the PBAF complex, which hinders REST’s ability to bind and inactivate its targets, leading to the upregulation of genes that promote melanoma brain metastases [6]. ARID2 also cooperates with BRD4 to maintain enhancer-promoter loops of these genes within the chromatin conformation, and the concurrent disruption of both ARID2 and BRD4 induces DNA damage [18]. However, the downstream transcriptional targets of ARID2 that contribute to GC metastasis remain poorly understood.

The Wnt/β-catenin signaling pathway is fundamental for maintaining normal physiological processes, including cell proliferation, differentiation, apoptosis, stem cell self-renewal, tissue homeostasis, and wound repair. Consequently, dysregulation of the Wnt/β-catenin signaling pathway acts as a crucial driver that promotes and sustains the malignant phenotypes of tumor cells [19,20]. Notably, the nuclear translocation of β-catenin enhances Wnt/β-catenin signaling activity, thereby promoting GC progression and lymph node metastasis [21]. The vitamin D receptor (VDR), a prominent member of the nuclear receptor family, exerts tumor-suppressive effects by regulating cell proliferation, apoptosis, and differentiation [22]. In ovarian cancer, VDR activation induces the deacetylation of β-catenin and inhibits its nuclear translocation, thereby potently antagonizing Wnt/β-catenin signaling and downregulating the expression of oncogenes [23]. In addition, VDR exhibits the potential to attenuate the malignant phenotypes of GC cells [24]. Despite these findings, the specific regulatory mechanisms of VDR in GC remain largely unexplored.

Vitamin D3 is a fat-soluble secosteroid prohormone that requires metabolic activation. In the classical pathway, vitamin D3 undergoes sequential hydroxylation to generate the active metabolite 1,25-dihydroxyvitamin D3 (1,25(OH)_2_D3, calcitriol), the principal high-affinity ligand of VDR. In addition to the classical pathway, *CYP11A1* can initiate alternative vitamin D3 metabolic pathways that generate multiple hydroxylated vitamin D derivatives, which may exert biological effects through VDR and/or other nuclear receptors [25]. These findings highlight the complexity of vitamin D metabolism and signaling. Upon binding calcitriol, VDR triggers the dissociation of corepressors and the recruitment of coactivators and mediator complexes, which further enlist epigenetic and chromatin remodeling machinery to reconfigure the nucleosomal landscape at VDR-binding sites, ultimately governing downstream signaling networks [26]. Despite the reported tumor-suppressive functions of VDR, it remains unclear whether ARID2 deficiency disrupts VDR signaling and whether calcitriol-mediated VDR activation can counteract Wnt/β-catenin-driven malignant phenotypes in GC.

Given the potent inhibitory effect of VDR on Wnt/β-catenin signaling, it is imperative to determine whether VDR acts as a key downstream effector in ARID2-mediated tumor suppression. In this study, we hypothesized that ARID2 deficiency decreases VDR transcription and enhances GC tumorigenesis by activating the Wnt signaling pathway. To test this hypothesis, we first investigated the functional role of ARID2 in GC metastasis and malignant progression. Mechanistically, we explored the regulatory crosstalk between ARID2 and VDR, specifically focusing on its impact on the Wnt/β-catenin signaling pathway. Ultimately, our study aimed to determine whether VDR activation by calcitriol could prevent β-catenin nuclear translocation and suppress Wnt signaling, thereby highlighting calcitriol as a promising therapeutic candidate for ARID2-deficient GC patients.

## Methods

### Cell culture and treatment

HGC-27 cells (RRID:CVCL_1279), SNU-601 cells (RRID:CVCL_0101), and AGS cells (RRID:CVCL_0139) were cultured in RPMI-1640 medium (Thermo Fisher, C11875500BT) supplemented with 10% fetal bovine serum (FBS) and incubated at 37°C with 5% CO_2_. MKN45 cells (RRID:CVCL_0434) were cultured in DMEM/F12 (Thermo Fisher, C11330500BT) containing 10% FBS under 5% CO_2_ conditions. Silencing and overexpression of ARID2 and VDR were performed by transfecting cells with specific shRNAs and overexpression plasmids, respectively. The corresponding sequences are listed in Supplementary Table S1, and knockdown efficiency was confirmed by western blotting. For treatment with calcitriol (MCE, HY-10002), the biologically active hormonal metabolite of vitamin D3, cells were incubated with 100 nM calcitriol prepared in DMSO for 48 h. The same final concentration of DMSO as in the calcitriol treatment group was administered to the vehicle control group.

### Cell-matrix adhesion assay

For the cell-matrix adhesion assay, cells were seeded at 5 × 10^4^ cells on 24-well plates precoated with collagen type I. After incubation for 1 h at 37°C, the wells were gently washed with PBS to remove any nonadherent cells. The remaining adherent cells were fixed with methanol for 15 min, after which the fixed cells were directly visualized and photographed using an inverted microscope and counted by ImageJ software.

### Wound healing assay

GC cells were seeded in 6-well plates. After 16 h, shaped wounds were scratched with a 10 μL pipette tip across each well. The cells were gently washed with PBS and cultured at 37°C in 5% CO_2_. The scratch zones were captured at 0 h and 12 h. The wound closure rate was calculated using ImageJ software.

### Transwell migration and invasion assays

A total of 4 × 10⁵ cells suspended in 200 μL of serum-free DMEM were seeded into the upper chambers of 24-well transwell inserts. For the invasion assay, the transwell inserts were precoated with Matrigel. The bottom wells were filled with 600 μL of DMEM containing 20% FBS. The remaining cells on the upper chamber were carefully removed using cotton swabs after 24 h. Migrated or invaded cells were then fixed with 4% paraformaldehyde and stained with crystal violet. Images were acquired using an inverted microscope and analyzed by ImageJ software.

### Quantitative real-time PCR (RT‒qPCR)

Cells were lysed with RNAiso Plus reagent (Takara, 9108). cDNA was obtained using the PrimeScript™ RT reagent Kit (Takara, RR037A). All RT‒qPCR experiments were performed using Taq Pro Universal SYBR qPCR Master Mix (Vazyme, Q712). The primers used for RT‒qPCR were designed in our laboratory and synthesized by Sangon Biotech (Shanghai, China). The primer sequences are listed in Supplementary Table S1. The relative expression levels of target genes were normalized to GAPDH and quantified using the 2^(−ΔΔCt) method.

### Western blotting

Whole cells were lysed in Co-IP/WB Tissue/Cell Lysis Buffer (Affinibody, AIWB-012) according to the manufacturer’s instructions. A bicinchoninic acid (BCA) protein assay kit (Beyotime, P0010) was used to determine protein concentrations. The primary antibodies used in this study are listed in Supplementary Table S2.

### Coimmunoprecipitation (Co-IP)

Cells were collected in Co-IP/WB Tissue/Cell Lysis Buffer (Affinibody, AIWB-012). GC cell lysates were incubated with the anti-ARID2 antibody or rabbit control IgG at 4°C overnight, followed by incubation with Pierce Protein A/G Magnetic Beads at 4°C for 2 h. HEK-293T cell lysates were incubated with the Flag beads overnight at 4°C. Subsequently, western blotting was performed using the indicated antibodies.

### Dual-luciferase assay

The luciferase reporter gene plasmid was transfected into control, shARID2 or OE-ARID2 GC cells, cotransfected with a pRL-TK vector as an internal control. At 72 h posttransfection, the cells were lysed, and the luciferase activities were measured following the manufacturer's instructions (Promega).

### Immunofluorescence (IF)

IF assays were performed as described previously [14]. Briefly, GC cells were fixed with 4% paraformaldehyde (PFA) for 20 min, permeabilized with 0.1% Triton X-100 for 10 min, and incubated with an anti-β-catenin antibody overnight at 4°C, followed by incubation with the corresponding CoraLite594 secondary antibody (Proteintech, SA00013-4) at a 1:500 dilution for 1 h at room temperature. Next, the cells were incubated with DAPI (Beyotime, P0131) for 5 min to visualize nuclei. Fluorescence images were acquired using a Leica DMi8 system.

### Peritoneal metastasis model

Six-week-old female BALB/c nude mice were obtained from Beijing Vital River Laboratory Animal Technology Co., Ltd., housed under specific-pathogen-free (SPF) conditions with access to food and water, and randomly allocated to groups prior to inoculation. A total of 1 × 10^6^ GC cells suspended in 100 μL of PBS/Matrigel mixture (1:1) were injected intraperitoneally into nude mice to establish the GC peritoneal metastasis model. For therapeutic experiments, initiated 7 days post-tumor cell implantation, calcitriol was administered orally at 3.75 μg/kg every 48 h. Tumor growth was monitored by bioluminescence imaging. After 28 days, the animals were euthanized, and the relative number of metastatic nodules in the peritoneum was counted.

### Immunohistochemistry (IHC) staining

The human GC tissue microarray was purchased from AiFang Biological (HN20250401). The sections were dewaxed and rehydrated, subjected to antigen retrieval, and incubated with primary antibodies at 4°C overnight. Subsequently, the sections were incubated with secondary antibodies at room temperature for 30 min. DAB (DAKO) was used for visualization. The IHC score was calculated as the sum of the staining intensity score (0, none; 1, weak; 2, moderate; 3, strong) and the proportion of positive cells (1, ≤ 25%; 2, 26-50%; 3, 51-75%; 4, ≥ 75%).

### Cleavage under targets and tagmentation (CUT&Tag) sequencing

CUT&Tag was processed using the Vazyme TD903 kit according to the manufacturer’s instructions. The samples were harvested and washed and then incubated with ConA beads, followed by incubation with the ARID2 antibody. The transposome was used to resuspend the samples and was then activated by magnesium ions, leading to the integration of the junction into nearby DNA. The DNA was extracted and subsequently amplified. Finally, DNA libraries were sequenced by HaploX (Shenzhen, China).

### RNA-seq

Lentivirus-mediated knockdown of ARID2 was performed in GC cells, followed by total RNA extraction utilizing TRIzol reagent (*n* = 3 per group). RNA purification, reverse transcription, library construction, and sequencing were conducted by HaploX (Shenzhen, China). The raw RNA-seq data were analyzed as previously described [18].

### pG-MNase CUT&RUN for qPCR

The Hyperactive pG-MNase CUT&RUN Assay Kit for qPCR (Vazyme, HD101-01) was used to conduct the CUT&RUN qPCR following the manufacturer’s instructions. In brief, 1 × 10^5^ GC cells were collected and bound to pretreated ConA beads. The cell-bead complex was treated with 1 µL of the ARID2 antibody at 4°C overnight. The following day, the complex was incubated with the pG-MNase enzyme at 4°C for 1 h. Following two rounds of DIG washing, the cell-bead complex was fragmented using CaCl_2_ solution for 1 h at 4°C. Then, the reaction was terminated, and the DNA was released, extracted, and subjected to qPCR. Spike-in DNA was used as a control. The primer sequences are listed in Supplementary Table S1.

### Survival analysis

Kaplan‒Meier survival curves were generated using the *survminer* R package. Progression-free survival was analyzed according to ARID2 mutation status in The Cancer Genome Atlas (TCGA)-STAD cohort, whereas overall survival was analyzed according to VDR expression in the Asian Cancer Research Group (ACRG) cohort. Furthermore, the hazard ratio (HR) and 95% confidence interval (CI) were calculated using a Cox proportional hazards regression model and visualized with the *forestploter* R package.

### Clinicopathological and Molecular Association Analyses

Normalized gene-expression and clinicopathological data were obtained from the GEO datasets GSE84437, the ACRG cohort (GSE62254), and GSE2685. In the GSE84437 cohort, *ARID2* expression was compared among patients classified according to N stage (N0-N3) and T stage (T1-T4). In the ACRG cohort, *VDR* expression was compared across clinical stages (I-IV) and molecular subtypes, including MSS/TP53-, MSS/TP53+, MSI, and EMT. In the GSE2685 cohort, patients were classified according to metastasis status, and the proportions of patients with low or high *VDR* expression were compared between the metastasis-negative and metastasis-positive groups.

### Statistical analysis

All data were analyzed using GraphPad Prism 8.0 software and expressed as the mean ± SD. Comparisons between two groups were performed using a two-tailed Student’s *t* test. For comparisons among three or more groups, a one-way analysis of variance (ANOVA) followed by Tukey’s post hoc test was applied. A *P* value < 0.05 was considered statistically significant.

## Results

### ARID2 deficiency correlates with lymph node metastasis and poor prognosis in GC

ARID2, a core subunit of the SWI/SNF chromatin remodeling complex, is recurrently mutated across multiple human cancer types. Analysis of TCGA datasets revealed that the mutation frequency of ARID2 is approximately 8% in GC patients (Fig. 1A). Further survival analysis demonstrated that ARID2 mutation was associated with a significantly shorter progression-free survival time in patients with lymph node metastasis (Fig. 1B-C). Mutations are largely missense or nonsense with few hotspots, which is consistent with a loss-of-function pattern (Fig. 1D). Notably, ARID2 protein levels were markedly downregulated in ARID2-mutant MKN74 GC cells compared to other ARID2-wild-type cells (Fig. 1E). These results suggest that ARID2 deficiency correlates with an increased risk of cancer metastasis, consequently conferring poor prognosis in GC. To validate that these observations were not restricted to TCGA datasets, we performed IHC staining on 80 human GC tissue samples (Fig. 1F). Consistent with the TCGA results, we found that ARID2 protein expression was significantly decreased in tumor tissues compared to paracancerous tissues (Fig. 1G, *P* < 0.0001). Furthermore, reduced ARID2 levels were significantly associated with advanced lymph node metastasis (N2-3 vs. N0-1) (Fig. 1H, *P* = 0.0095). To further validate these findings in an independent cohort, we evaluated ARID2 expression across N and T stages in the GSE84437 cohort. The results consistently revealed a downregulation of ARID2 in advanced N stages (Fig. S1A). Similarly, patients in more advanced T stages (T2-T4) exhibited a trend toward decreased expression compared to those in the T1 stage, although statistical significance was not achieved (Fig. S1B). Collectively, these results indicate that ARID2 deficiency is a key contributor to GC progression, potentially via its involvement in tumor cell metastasis.

**Fig. 1:**
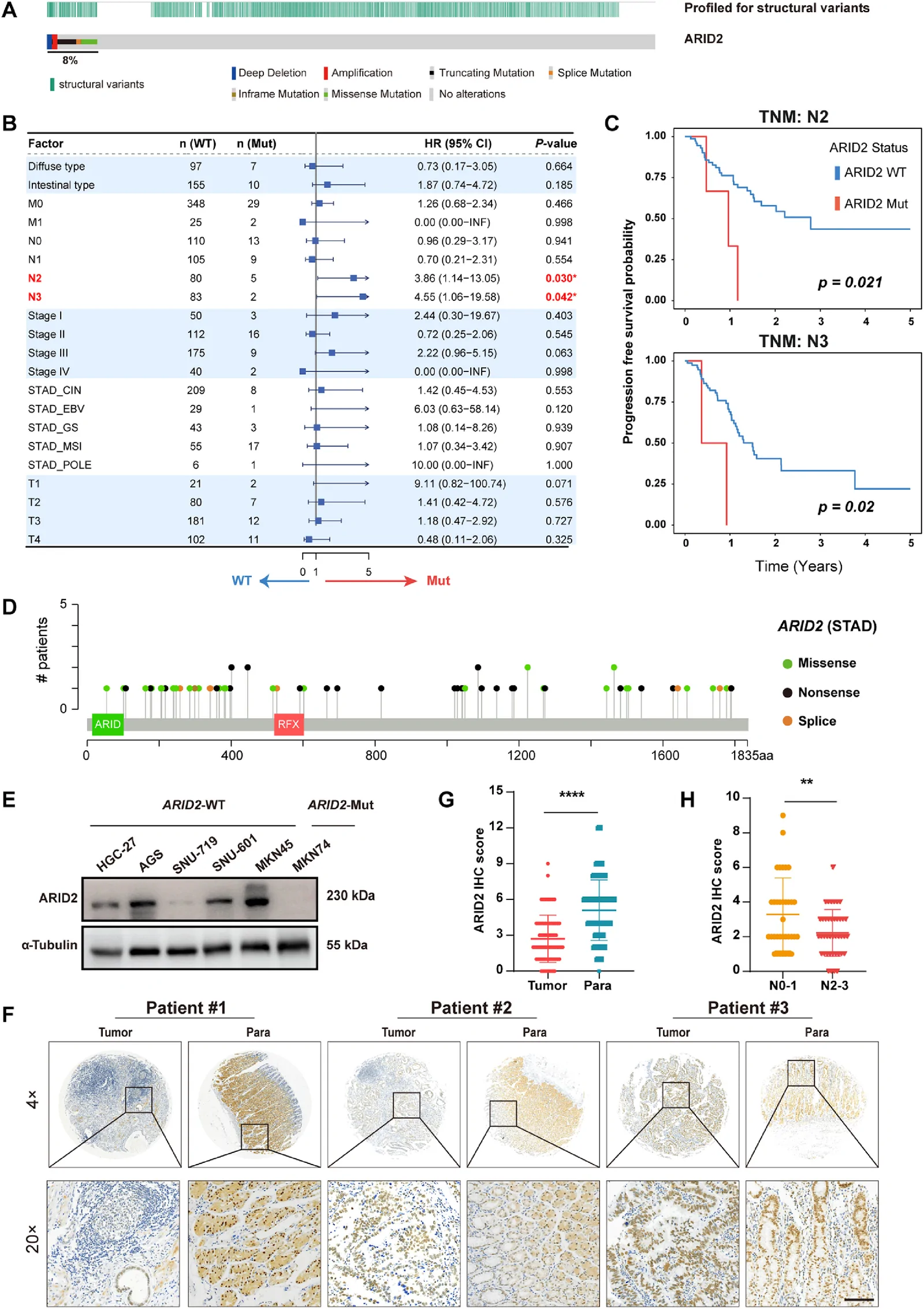
**ARID2 deficiency may drive GC progression and metastasis.** (A) Alteration frequency of *ARID2* in TCGA-STAD. (B) Forest plot showing subgroup analysis of progression-free survival based on *ARID2* mutation status across clinicopathological categories in TCGA-STAD. (C) Survival analysis of *ARID2* mutation for progression-free survival in patients with N2 and N3 stages in the TCGA-STAD dataset. (D) Lollipop plot showing the distribution and types of *ARID2* mutations across its protein domains in TCGA-STAD. (E) Protein levels of ARID2 in ARID2 wild-type and mutant GC cells (*n* = 3). (F) Representative ARID2 IHC staining images of tumor tissues and paracancerous tissues in GC microarrays. Scale bar: 100 μm. (G) ARID2 IHC scores of the indicated tissues (*n* = 80 per group). (H) IHC scores of ARID2 in tumor tissues stratified by N stage (N0-1, *n* = 35; N2-3, *n* = 41). Para: paracancerous tissues. ns, not significant; * *P* < 0.05, ** *P* < 0.01, *** *P* < 0.001, **** *P* < 0.0001.

### Downregulation of ARID2 drives GC metastasis in vitro and in vivo

To investigate the biological function of ARID2 in GC, two independent shRNAs (shARID2 #1 and #2) were used to silence ARID2 in GC cells (Fig S2A-D). We found that ARID2 knockdown significantly promoted cell-matrix adhesion in both HGC-27 and SNU-601 cells (Fig. 2A-B, Fig. S2E-F). Transwell assays revealed that ARID2 knockdown promoted the migration and invasion of GC cells (Fig. 2C-D, Fig. S2G-H). Consistently, wound healing assays showed that ARID2 knockdown increased the motility of GC cells (Fig. 2E-F, Fig. S2I-J). In an *in vivo* peritoneal metastasis model, ARID2-knockdown MKN45 cells generated a substantially higher macroscopic tumor burden in the peritoneal cavity, indicating enhanced metastatic colonization and growth (Fig. 2G-I). Together, these results establish ARID2 as a critical suppressor of GC malignancy, restraining tumor cell adhesion, motility, and metastatic dissemination.

**Fig. 2:**
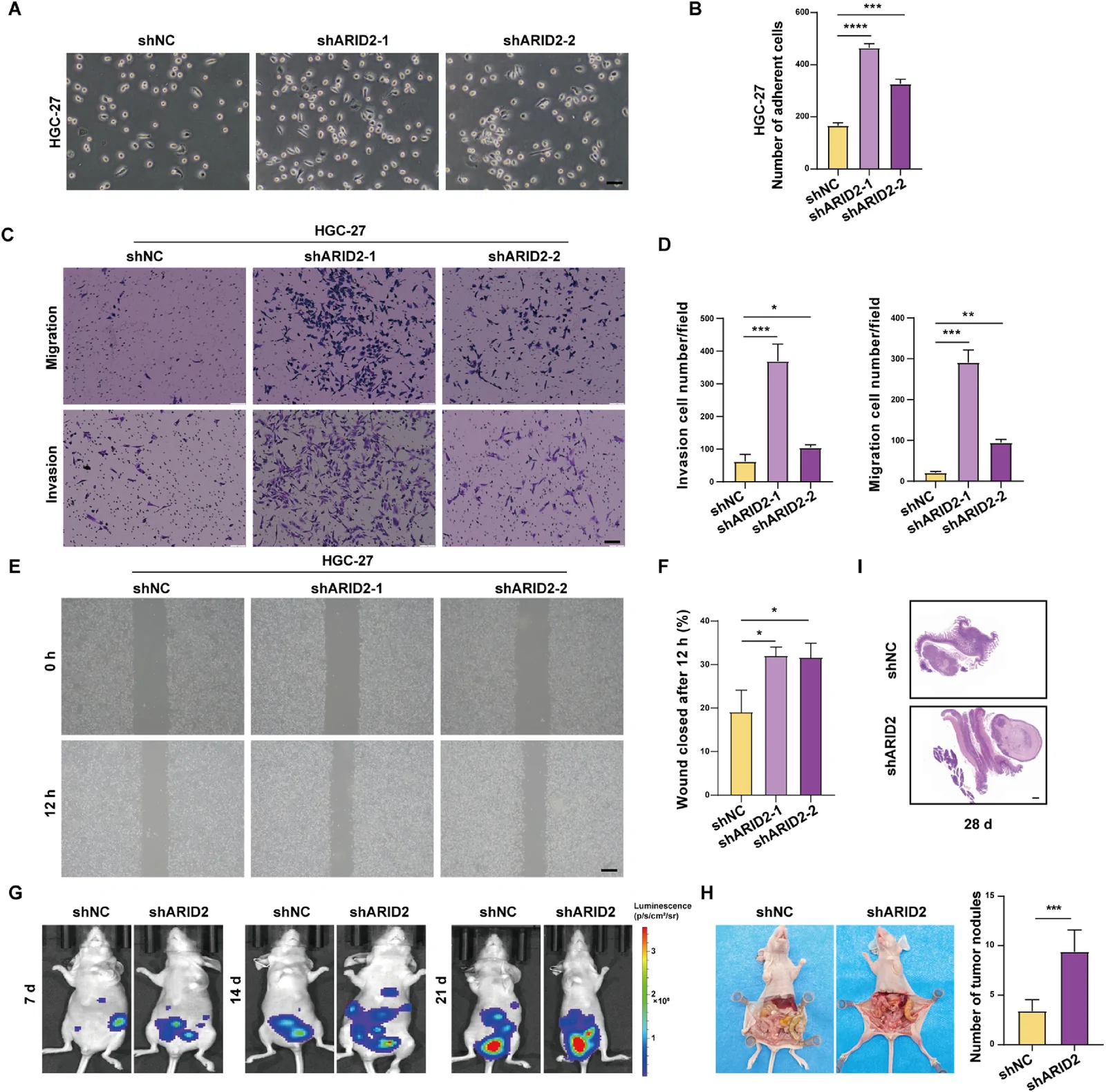
**Downregulation of ARID2 promotes cell adhesion, migration, and invasion in GC cells and accelerates tumor progression.** (A, B) The effects of ARID2 knockdown on the adhesive ability of GC cells were examined through the cell-matrix adhesion assay (scale bar = 100 μm, *n* = 3). (C) Transwell migration and invasion assays in ARID2 knockdown groups (scale bar = 200 μm, *n* = 3). (D) Results of the transwell assay. (E) Representative images of the wound healing assay using HGC-27 cells transduced with shARID2-1, shARID2-2 or shNC (scale bar = 200 μm). (F) Statistical analysis of the wound healing assay in HGC-27 cells (*n* = 3). (G) Tumor formation in the peritoneum was monitored by bioluminescence imaging after peritoneal injection of MKN45 cells. (H) Representative macroscopic findings of established peritoneal nodules after peritoneal injection of MKN45 cells. The total number of established peritoneal nodules was compared between the shARID2 and shNC groups (*n* = 5). (I) Representative H&E images of established peritoneal nodules after peritoneal injection of MKN45 cells (scale bar = 200 μm). ns, not significant; * *P* < 0.05, ** *P* < 0.01, *** *P* < 0.001, **** *P* < 0.0001.

### ARID2 deficiency activates Wnt/β-catenin signaling to promote GC progression

To elucidate how ARID2 deficiency influences the functions of GC cells during disease progression, we performed RNA-Seq analysis on HGC-27 cells with ARID2 knockdown. Numerous differentially expressed genes (DEGs) were identified between the negative control (shNC) and ARID2-knockdown (shARID2) cells. Gene Ontology (GO) and Kyoto Encyclopedia of Genes and Genomes (KEGG) enrichment analyses revealed that the upregulated DEGs were significantly enriched (*p* < 0.001) in extracellular matrix organization, cell adhesion, growth regulation, and the Wnt signaling pathway (Fig. 3A, Fig. S2K). Consistent with the known roles of oncogenic Wnt/β-catenin signaling in promoting ECM expression and tumor growth [27], our transcriptome data showed that multiple Wnt pathway target genes, including *Wnt6, Wnt5A, TCF4, FZD7, DAAM1*, and *CCND1*, were significantly upregulated in the shARID2 group (Fig. 3B). RT‒qPCR further confirmed significantly increased expression of Wnt signaling-associated genes (*CCND1, c-MYC*, and *TCF4*) in ARID2-knockdown cells compared to controls (Fig. 3C-D), reinforcing the involvement of the Wnt signaling pathway in ARID2-deficient gastric carcinogenesis. To assess the activation of the Wnt signaling pathway at the protein level, we examined the nuclear translocation of β-catenin in GC cells. IF analysis demonstrated that β-catenin was primarily localized in the nucleus of ARID2-knockdown GC cells (Fig. 3E). Consistently, ARID2 knockdown resulted in increased levels of β-catenin and several Wnt downstream proteins (including c-Myc and cyclin D1) (Fig. 3F-G). Altogether, these results indicate that ARID2 deficiency activates Wnt/β-catenin signaling and contributes to the malignant progression of GC.

**Fig. 3:**
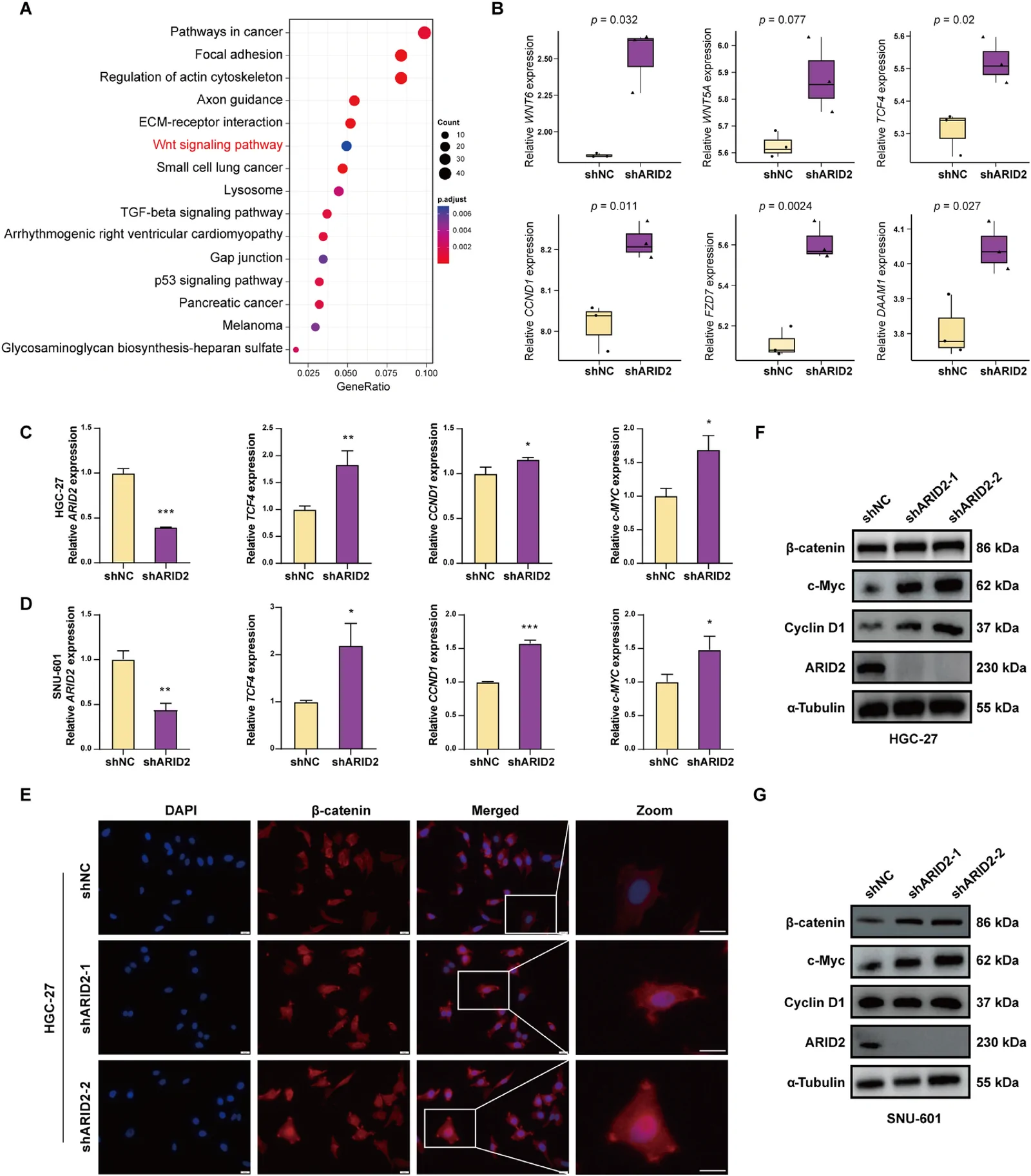
**Downregulation of ARID2 activates the Wnt/β-catenin signaling pathway.** (A) KEGG pathway analysis of upregulated DEGs following ARID2 knockdown. (B) Box plots depicting Wnt target gene expression identified by RNA-seq. (C, D) Wnt target gene expression changes were verified by RT‒qPCR in GC cells (*n* = 3). (E) Representative images of cells stained for β-catenin (red) and DAPI for nuclei (blue) (scale bar = 20 μm). (F, G) Western blotting for β-catenin, c-Myc, and cyclin D1 protein levels in GC cells after ARID2 knockdown (*n* = 3). ns, not significant; * *P* < 0.05, ** *P* < 0.01, *** *P* < 0.001, **** *P* < 0.0001.

### ARID2 interacts with VDR and activates the transcription of VDR in GC cells

ARID2 regulates gene expression through differential genomic binding and/or recruitment of TFs. To further elucidate the possible mechanism by which ARID2 deficiency drives GC progression and malignancy, we performed integrative analyses of CUT&Tag seq and RNA seq. A total of 54,544 ARID2-specific binding peaks were identified (Fig. S2L). Then, we used Lisa [28] to predict the TFs associated with genes upregulated in ARID2 knockdown cells. To identify GC progression ARID2 transcriptional targets, we integrated results from (i) the promoter-proximal ARID2 CUT&Tag peaks, (ii) HOMER-predicted enriched TFs (*p* < 0.001), and (iii) LISA-predicted TFs. Strikingly, VDR was the only gene shared across all three datasets (Fig. 4A-B). CUT&RUN qPCR further validated ARID2 enrichment at the *VDR* promoter, which was markedly reduced upon ARID2 knockdown (Fig. 4C). Consistently, ARID2 knockdown significantly suppressed *VDR* promoter activity (Fig. 4D, *P* = 0.0003), whereas ARID2 overexpression enhanced it (Fig. 4E, *P* = 0.008).

**Fig. 4:**
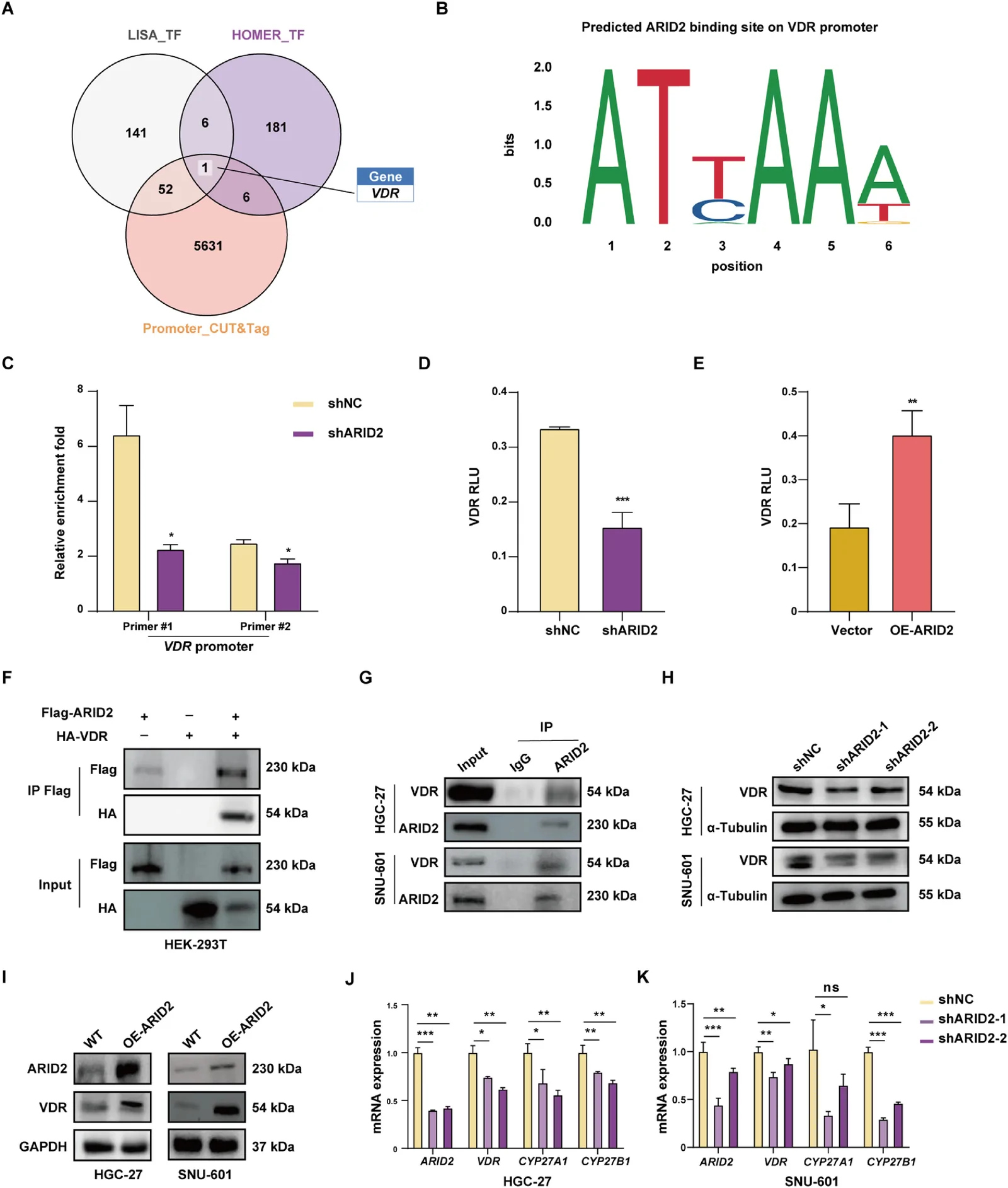
**ARID2 activates VDR transcription and physically interacts with the VDR protein.** (A) Venn diagram showing the overlap of promoter binding sites, HOMER-predicted TFs, and LISA-predicted TFs from RNA-seq and CUT&Tag datasets. (B) The predicted ARID2 binding motif on the VDR promoter. (C) CUT&RUN qPCR was performed in HGC-27 cells to identify the enrichment of ARID2 in the *VDR* promoter region (*n* = 3). (D, E) Analysis of VDR promoter activity in response to shARID2 and OE-ARID2. VDR promoter luciferase reporter constructs were transfected into HGC-27 cells, and luciferase activity was measured (*n* = 3). (F) HEK-293T cells were transfected with Flag-tagged ARID2 and HA-tagged VDR and subjected to Co-IP using an anti-Flag antibody (*n* = 3). (G) Co-IP assays confirmed the interaction between ARID2 and VDR in GC cells (*n* = 3). (H) Western blot analysis was performed to measure the protein levels of VDR in GC cells transfected with shARID2 (*n* = 3). (I) Western blot analysis was performed to measure the protein levels of VDR in HGC-27 cells overexpressing ARID2 (*n* = 3). (J, K) RT‒qPCR analysis was performed to measure the expression of VDR target genes in GC cells transfected with shARID2 (*n* = 3). ns, not significant; * *P* < 0.05, ** *P* < 0.01, *** *P* < 0.001, **** *P* < 0.0001.

To determine whether ARID2 physically interacts with VDR, we performed Co-IP assays using protein extracts from HEK-293T and multiple GC cells. These experiments confirmed the interaction between ARID2 and VDR (Fig. 4F-G). Notably, western blot analysis showed that the protein levels of VDR were decreased following ARID2 knockdown and increased upon ARID2 overexpression (Fig. 4H-I). ARID2 knockdown also reduced the expression of well-known target genes of VDR (Fig. 4J-K), consistent with our transcriptome findings (Fig. S3A). Moreover, both the mRNA and protein levels of Wnt target genes were upregulated upon VDR knockdown and were effectively restored upon VDR overexpression (Fig. S3B-E), suggesting that VDR mediates the Wnt signaling pathway activation induced by ARID2 deficiency. Collectively, these results demonstrate that ARID2 interacts with and transcriptionally activates VDR and that disruption of ARID2-VDR signaling contributes to Wnt signaling pathway activation in GC.

### VDR activation preferentially suppresses the Wnt/β-catenin signaling driven by ARID2 deficiency in GC

VDR belongs to the steroid receptor family and contains a highly conserved NH2-terminal DNA binding domain (DBD) and the more variable COOH-terminal ligand binding domain (LBD) [29]. VDR has significant implications for cancer progression by regulating cancer stemness, cell differentiation, and proliferation [30]. In line with its potential tumor-suppressive role, univariate Cox analysis of the ACRG dataset identified lower VDR expression, along with clinical variables including age, tumor grade, gender, and N-stage, as significant risk factors for poor prognosis in GC patients (Fig. S4A). Kaplan‒Meier analysis further demonstrated that lower VDR expression was significantly correlated with shorter overall survival, underscoring the prognostic importance of VDR in GC (Fig. S4B). Within the same ACRG cohort, we observed that VDR expression was significantly lower in stage III and IV tumors than in stage I tumors (Fig. S4C). Furthermore, when stratifying these GC cases by molecular subtypes, VDR expression was strikingly diminished in the epithelial–mesenchymal transition (EMT) subtype, a phenotype notorious for high aggressiveness and poor outcomes, compared to the MSS/TP53-, MSS/TP53+, and MSI subtypes (Fig. S4D). Finally, clinical data from an additional dataset (GSE2685) corroborated these findings, demonstrating that the proportion of patients with low VDR expression was markedly enriched in the metastasis-positive group relative to the metastasis-negative group (Fig. S4E). Collectively, these data strongly indicate that within gastric tumors, VDR downregulation is closely linked to disease progression, a malignant phenotype, and increased metastatic potential.

To determine whether VDR is functionally involved in the Wnt/β-catenin signaling pathway activation driven by ARID2 deficiency, we overexpressed VDR in ARID2-knockdown GC cells. Notably, VDR overexpression markedly reduced the nuclear translocation of β-catenin (Fig. 5A). In parallel, ARID2 knockdown enhanced both the mRNA and protein levels of key Wnt target genes, such as β-catenin, c-MYC, and TCF4, whereas these effects were all effectively reversed by VDR overexpression (Fig. 5B-C). These findings indicate that the aberrant Wnt/β-catenin signaling promoted by ARID2 deficiency can be attenuated by VDR restoration.

**Fig. 5:**
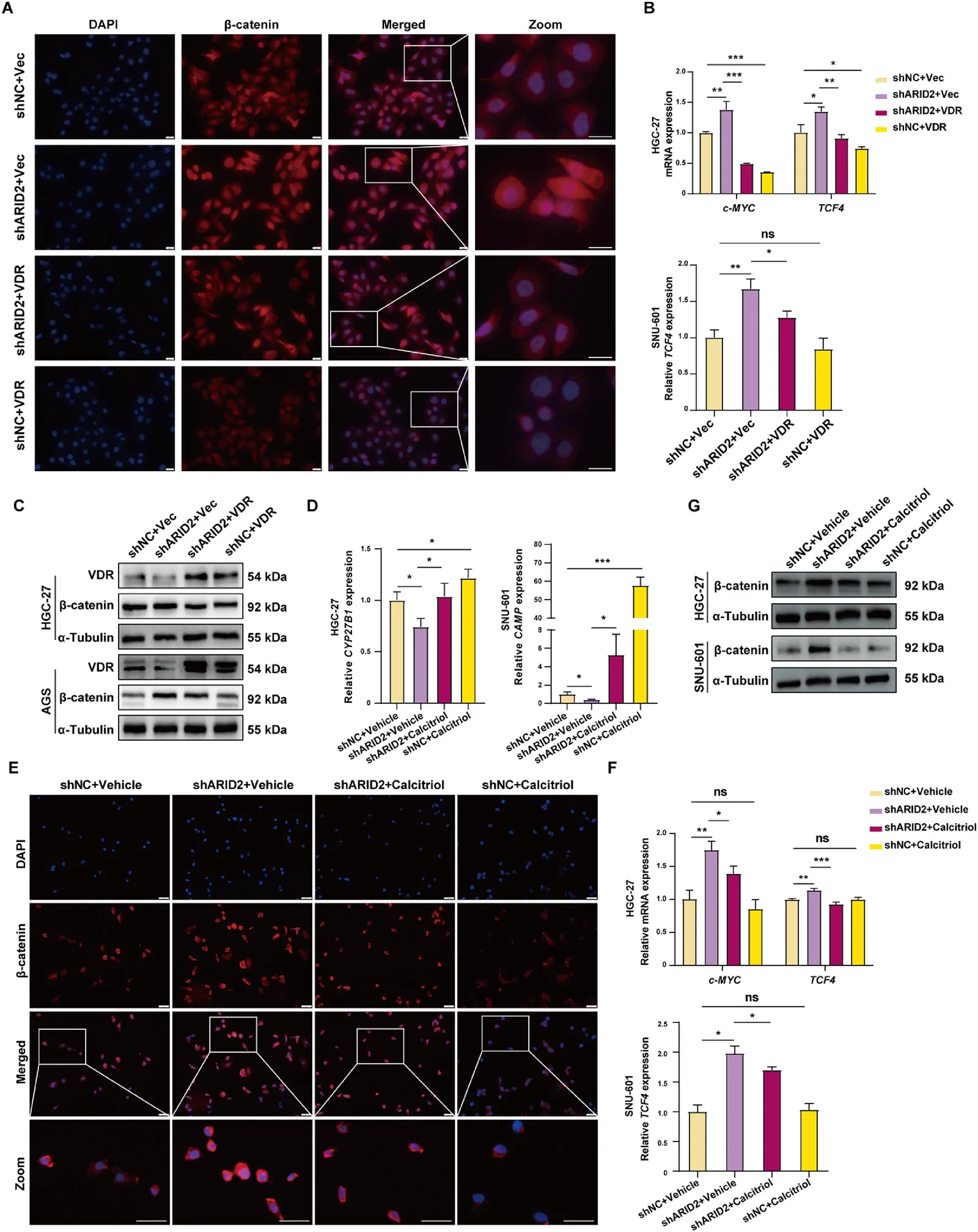
**VDR activation inhibits the Wnt/β-catenin signaling induced by ARID2 knockdown.** (A) IF assay showing the localization of β-catenin in AGS cells following ARID2 knockdown and/or VDR overexpression (scale bar = 20 μm, *n* = 3). (B, C) The mRNA and protein levels of Wnt target genes in GC cells with ARID2 knockdown and/or VDR overexpression (*n* = 3). (D) RT‒qPCR analysis of VDR target genes in GC cells with ARID2 knockdown and/or calcitriol treatment (*n* = 3). (E) IF assay showing the localization of β-catenin in HGC-27 cells following ARID2 knockdown and calcitriol treatment (scale bar = 50 μm, *n* = 3). (F, G) The mRNA and protein levels of Wnt target genes in ARID2-knockdown GC cells treated with calcitriol (*n* = 3). ns, not significant; * *P* < 0.05, ** *P* < 0.01, *** *P* < 0.001, **** *P* < 0.0001.

Calcitriol regulates diverse physiological and pathological pathways through VDR activation [26]. The mRNA expression of VDR target gene was decreased following VDR knockdown in GC cells. Notably, this reduction was abolished upon calcitriol treatment (Fig. S5A-D). Consistently, *CYP27B1* and cathelicidin antimicrobial peptide (*CAMP*) induction, the major VDR target genes serving as the control for calcitriol activity, was observed in both the shARID2 and shNC groups upon calcitriol treatment (Fig. 5D). The enhanced β-catenin nuclear translocation triggered by ARID2 knockdown was also reversed upon calcitriol treatment (Fig. 5E). Furthermore, the mRNA and protein levels of Wnt target genes supported the same conclusion that calcitriol effectively counteracts Wnt signaling pathway activation driven by ARID2 knockdown (Fig. 5F-G). Together, these results indicate that VDR activation preferentially inhibits the Wnt/β-catenin signaling pathway in GC cells with ARID2 knockdown, thereby counteracting the protumorigenic consequences of ARID2 deficiency.

### VDR activation preferentially suppresses the malignant progression of ARID2-knockdown GC

To investigate whether VDR activation influences tumor metastasis induced by the downregulation of ARID2, we first assessed whether VDR affects GC cell motility. Both VDR overexpression and calcitriol treatment significantly alleviated the effects of ARID2 silencing on the adhesion ability of GC cells (*P* < 0.01), a critical step for cancer metastasis (Fig. S6A-D). To further validate this effect *in vivo*, we established a peritoneal metastasis model using MKN45-derived xenografts expressing shNC+Vector, shNC+VDR, shARID2+Vector, and shARID2+VDR (Fig. 6A). Tumor growth was monitored using an *in vivo* imaging system (IVIS). VDR overexpression markedly reduced the tumor burden and the number of metastatic nodules in both ARID2-NC and ARID2-depleted xenografts (Fig. 6B-C). Additionally, body weight showed no significant differences among the different experimental groups (Fig. S6E).

**Fig. 6:**
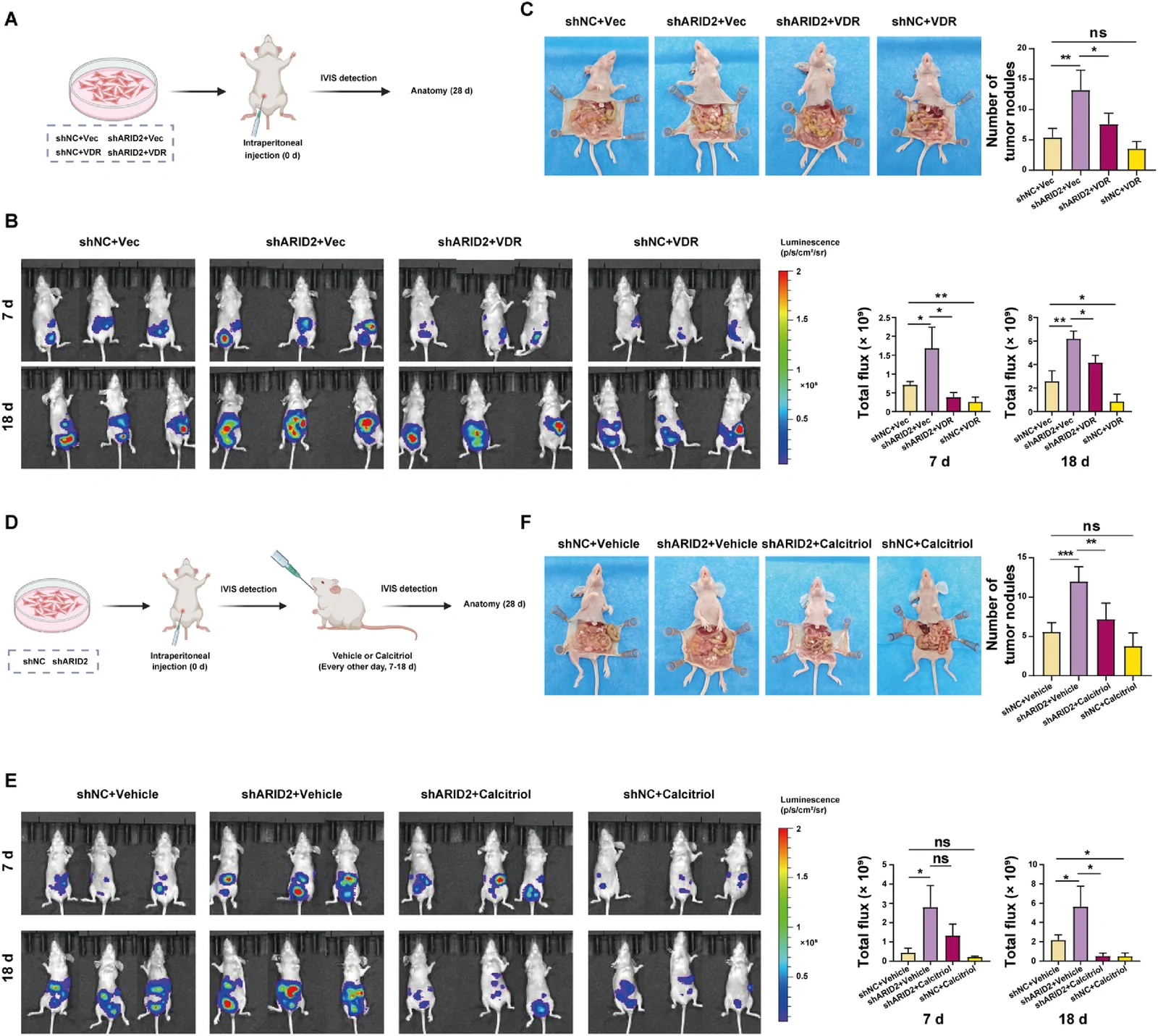
**VDR overexpression and treatment with the VDR activator calcitriol attenuate the metastatic potential induced by ARID2 knockdown.** (A) Flowchart of the mouse tumor model in which GC cells were intraperitoneally injected into nude mice. (B) The progression of GC xenografts was monitored through bioluminescence imaging, and representative images were displayed. (C) The morphological characteristics of peritoneal tumor xenografts and the number of metastases in peritoneal foci were evaluated (*n* = 5). (D) Schematic of the *in vivo* experimental design in which mice were injected with GC cells and treated with oral calcitriol. (E) IVIS images of individual mice bearing intraperitoneal tumors treated with either calcitriol or vehicle control. (F) The morphological characteristics of peritoneal tumor xenografts and statistical analysis of the number of metastases in peritoneal foci of different groups (*n* = 5). ns, not significant; * *P* < 0.05, ** *P* < 0.01, *** *P* < 0.001, **** *P* < 0.0001.

For therapeutic assessment, the mice were intraperitoneally injected with 1 million MKN45 cells under different treatments: shNC+Vehicle, shARID2+Vehicle, shNC+Calcitriol, and shARID2+Calcitriol (Fig. 6D). IVIS imaging revealed that ARID2 knockdown significantly increased the fluorescence intensity and the number of metastatic nodules compared with those in the control group; however, calcitriol treatment substantially reduced both the fluorescence intensity and the number of metastatic nodules in the shARID2+Calcitriol group compared to the shARID2+Vehicle group (Fig. 6E-F), again without affecting the body weight of tumor-bearing mice (Fig. S6F). In addition, calcitriol treatment significantly reduced the fluorescence intensity on day 18 and slightly decreased the number of metastatic nodules compared with the shNC+Vehicle group (shNC+Calcitriol vs. shNC+Vehicle, Fig. 6E-F).

Taken together, these findings demonstrate that VDR activation exerts superior antitumor effects in ARID2-deficient GCs. Therefore, pharmacological activation of VDR signaling with calcitriol might serve as an effective therapeutic strategy for treating metastatic GC driven by ARID2 deficiency.

### Reduced ARID2 and VDR levels correlate with advanced regional lymph node metastasis in GC patients

To further characterize the roles of ARID2 and VDR in GC progression, we performed IHC assays to examine their protein levels in GC tissues and adjacent normal tissues (Fig. 7A, Fig. S7A). Consistent with the reduced ARID2 expression, VDR levels were also significantly downregulated in GC tissues compared with adjacent nontumor tissues, as determined by IHC (Fig. S7B). Notably, GC patients with high ARID2 expression also exhibited higher VDR expression (Fig. 7B, Fig. S7C). Spearman correlation analysis indicated a significant correlation between the IHC scores of ARID2 and VDR in GC tissues (Fig. 7C). These results emphasize the role of ARID2 as a positive regulator of VDR expression in GC. Moreover, the IHC scores of VDR in the N2-3 group were significantly lower than those in the N0-1 group (Fig. S7D). The distribution of clinical stage and N stages among patients with different protein levels of ARID2 and VDR is shown in Fig. 7D. Specifically, low ARID2 and VDR expression levels were strongly correlated with higher lymph node metastasis and more advanced clinical stages of GC. Collectively, our findings indicate that reduced ARID2 and VDR expression correlates with aggressive disease features, suggesting that their coordinated activity plays a critical role in GC metastasis.

**Fig. 7:**
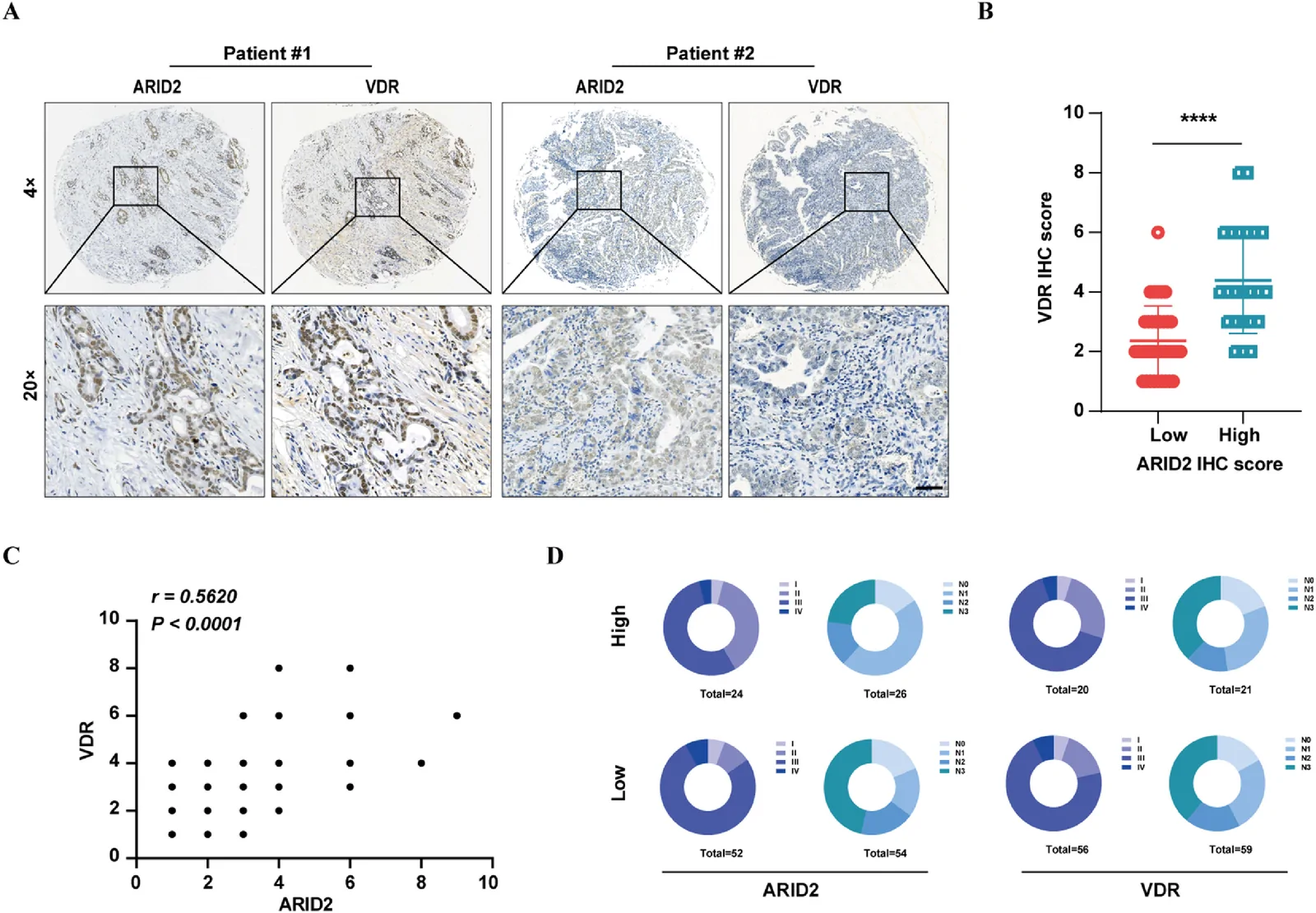
**Low expression of both ARID2 and VDR correlates with advanced clinical features of GC.** (A) Representative IHC staining of ARID2 and VDR in GC samples (scale bar = 50 μm, *n* = 80). (B) Comparison of VDR IHC scores in GC patients with low (n = 36) and high (n = 23) ARID2 expression. (C) Spearman correlation analysis between ARID2 and VDR in GC tissues. (D) The distribution of lymph node metastasis and clinical stage features in the low and high ARID2 and VDR groups. * *P* < 0.05, ** *P* < 0.01, *** *P* < 0.001, **** *P* < 0.0001.

## Discussion

SWI/SNF complexes play a tumor-suppressor role and are mutated significantly more often than any other chromatin remodeler in distinct cancer types [[31], [32], [33]]. Mutations of SWI/SNF subunits observed in cancer are typically loss-of-function and disrupt complex assembly, ATP-dependent chromatin remodeling, and chromatin binding [34]. As a subunit of the SWI/SNF chromatin remodeling complex, ARID2 is frequently mutated in certain types of cancer [5]. Although ARID2 has been shown to exhibit a tumor-suppressive function in gastric carcinogenesis [11], the mechanism by which ARID2 regulates GC progression and metastasis remains poorly understood. Identifying patients with ARID2 mutations in clinical practice may offer opportunities for personalized therapeutic strategies.

GC is one of the most common gastrointestinal malignant tumors, and metastasis of cancer cells is associated with poor prognosis. In this study, we found that ARID2 mutation or downregulation is strongly associated with advanced lymph node metastasis in GC. Our functional assays demonstrated that ARID2 knockdown significantly enhanced GC cell invasion and metastasis. Fundamentally, ARID2 maintains normal transcriptional programs as a chromatin remodeler. Consequently, ARID2 dysregulation likely triggers chromatin disorganization, endowing GC cells with the plasticity to aberrantly activate pathways that drive metastasis. Furthermore, recent evidence has shown that ARID2 mutations undergo statistically significant positive selection in normal gastric glands, indicating that ARID2 deficiency may initiate gastric carcinogenesis [12]. Ultimately, ARID2 deficiency may govern the specific regulatory mechanisms of metastasis in GC. Thus, ARID2-mutated GC emerges as a distinct molecular subtype, providing the fundamental basis for clinical precision interventions.

The Wnt signaling pathway serves as a key regulator of oncogenesis by governing cell cycle progression, apoptosis, EMT, stemness, the tumor immune microenvironment, and vascular homeostasis, thereby playing a central role in the pathogenesis of diverse types of cancer [35]. Accumulated cytoplasmic β-catenin translocates to the nucleus, where it interacts with the T-cell factor (TCF) family TFs and coactivators to activate canonical Wnt signaling and induce the transcription of downstream targets, including *MYC* and *CCND1* [19,36]. Noncanonical Wnt signaling mediated by WNT5a drives stem cell maintenance and invasion through the coreceptor ROR1 in prostate cancer [37]. Inactivation of Wnt signaling axes has been shown to prevent lung tumor metastasis and prolong survival in animal models [38]. By promoting cell proliferation and invasion and inhibiting cell death, increased *CTNNB1* transcription enhances peritoneal dissemination in GC cells [39]. In our study, downregulation of ARID2 activated Wnt signaling in GC, leading to β-catenin nuclear translocation and the subsequent activation of a prometastatic transcriptional program, as evidenced by increased mRNA and protein levels of c-MYC and cyclin D1. Although the role of the Wnt pathway in cancer progression has been well documented, our findings provide novel evidence that ARID2 restrains Wnt/β-catenin signaling, thereby inhibiting GC invasion and metastasis.

Chromatin remodelers often contain DNA-binding and/or chromatin reader domains, which modulate the genomic occupancy of TFs and complex targeting and activity [40]. These findings prompted us to hypothesize that in ARID2-deficient GC, the loss of the chromatin remodeler ARID2 disrupts normal regulatory interactions with TFs at Wnt-related *cis*-regulatory sequences, thereby leading to Wnt hyperactivation. Notably, our results suggest that VDR serves as a pivotal mediator in this regulatory axis. We found that ARID2 is enriched at the VDR promoter region and enhances VDR promoter activity, thereby positively regulating VDR expression. VDR is a nuclear receptor TF that plays an important role in mediating multiple antitumor effects [22,41]. The downregulation of VDR expression has been implicated in the progression of various aggressive cancers [42]. VDR exerts an antitumor effect in hepatocellular carcinoma, and its nuclear translocation enhances the effectiveness of immunotherapy [43]. VDR status is essential for the maintenance of intestinal homeostasis, and its deletion accelerates the transformation of chronic intestinal inflammation-induced cancer [44]. Mechanistically, VDR can interact with β-catenin, thereby inhibiting its nuclear translocation and the expression of β-catenin target genes in colorectal cancer [45]. Consistent with these observations, we demonstrated that overexpression of VDR in ARID2-knockdown GC cells significantly inhibited β-catenin nuclear translocation and reduced the expression of Wnt signaling target genes. Moreover, patients with low expression of both ARID2 and VDR in tumor tissues exhibited more aggressive clinical features and a trend toward worse overall survival. Specifically, our findings reveal that the VDR/Wnt/β-catenin axis plays a critical role in the metastasis of ARID2-deficient GC.

Calcitriol, the hormonally active metabolite of vitamin D3, binds to VDR and forms a functional transcription complex by dimerizing with the Retinoid X Receptor, its primary partner. This ligand-dependent VDR-RXR heterodimer complex binds specifically to vitamin D response elements located in the promoter regions of target genes, thereby activating or repressing their expression [46]. Extensive studies have established the pleiotropic therapeutic potential of calcitriol in various malignancies, including colorectal, prostate, and breast cancers [[47], [48], [49]]. In line with these findings, our study further expanded the clinical utility of calcitriol, identifying it as a highly effective targeted therapeutic strategy for ARID2-deficient GC. Calcitriol treatment specifically targeted VDR in ARID2-knockdown GC cells, thereby restoring the expression of canonical VDR target genes and attenuating hyperactivation of the Wnt/β-catenin signaling pathway. Notably, calcitriol treatment potently inhibited the invasiveness and metastatic capacity of ARID2-knockdown GC cells. This antimetastatic effect was consistently observed in established cell lines *in vitro* and further validated in xenograft mouse models *in vivo*. Mechanistically, calcitriol-mediated activation of VDR suppresses Wnt/β-catenin signaling by preventing the nuclear translocation of β-catenin in the context of ARID2 deficiency. Our findings support the therapeutic potential of calcitriol for suppressing metastasis in ARID2-deficient GC.

In this study, calcitriol attenuated malignant phenotypes *in vitro* and suppressed peritoneal metastasis *in vivo*. However, we acknowledge that calcitriol-associated hypercalcemia and hypercalciuria remain important dose-limiting safety concerns. In addition, the use of ARID2-knockout animal models would provide a more rigorous *in vivo* evaluation of the ARID2-VDR axis. Therefore, future studies should incorporate ARID2-knockout models, dose-response analyses, and systematic safety monitoring to define the therapeutic window of calcitriol in ARID2-deficient GC. Large-scale prospective clinical studies are also warranted to validate these findings and determine the optimal and safe calcitriol regimen for this patient subgroup. Our current study provides a solid mechanistic rationale for these future investigations.

## Conclusion

In summary, our study demonstrates that ARID2 deficiency promotes GC cell-matrix adhesion and metastasis by enhancing the nuclear translocation of β-catenin and activating the Wnt/β-catenin signaling pathway. We elucidate a novel regulatory role for ARID2 in binding the *VDR* promoter, thereby modulating *VDR* transcription and target gene expression. Most importantly, our findings indicate that calcitriol-mediated VDR activation attenuates GC progression under ARID2-deficient conditions by inhibiting hyperactivation of the Wnt/β-catenin signaling pathway. Therefore, targeting the VDR/Wnt/β-catenin axis may represent a potential therapeutic strategy for ARID2-deficient GC patients.

## Ethical and legal declarations

All animal experiments were approved by the Animal Care and Use Committee of Huazhong University of Science and Technology (2025-5320). The human gastric cancer tissue microarray project was reviewed and approved by the Biological Science and Technology Ethics Review Committee of Hunan Aifang Biotechnology Co., Ltd. (HN20250401).

## Availability of data and materials

The raw data for RNA-seq and CUT&Tag have been deposited in the NCBI Sequence Read Archive database under the accession number PRJNA1391065.

## Funding

This work was supported by the fundamental research funds for the central universities, HUST (No. 2021GCRC073 to Ximiao He), the Natural Science Foundation of Chongqing (No. CSTB2024NSCQ-LZX0015 to Tao Wang, CSTC2020JCYJ-MSXMX0181 to Biying Liu), the National Natural Science Foundation of China (NSFC No. 82002582 to Biying Liu).

## CRediT authorship contribution statement

**Biying Liu:** Writing – original draft, Validation, Supervision, Project administration, Investigation, Funding acquisition, Data curation. **Zhaole Chu:** Validation, Methodology, Conceptualization. **Yixian Fan:** Validation, Software, Methodology. **Ye Tian:** Investigation, Formal analysis. **Jing Jin:** Writing – review & editing, Formal analysis. **Tao Wang:** Writing – review & editing, Funding acquisition, Formal analysis. **Ximiao He:** Writing – review & editing, Funding acquisition, Formal analysis, Data curation, Conceptualization.

## Declaration of competing interest

The authors declare that they have no known competing financial interests or personal relationships that could have appeared to influence the work reported in this paper.
