# Supplementary material for: Calcitriol alleviates ARID2-deficient gastric cancer metastasis via inhibition of the Wnt/β-catenin signaling pathway
Source: Transl Oncol. 2026 Sep 22;73:103048. doi: 10.1016/j.tranon.2026.103048 (PMC13633874; doi:10.1016/j.tranon.2026.103048)

Figure 1E:

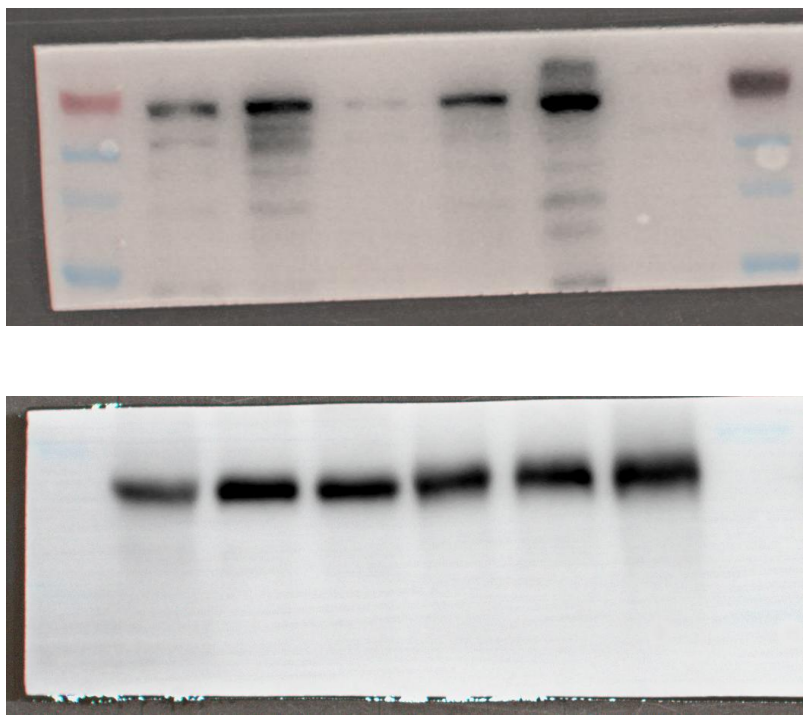

Figure 3F:

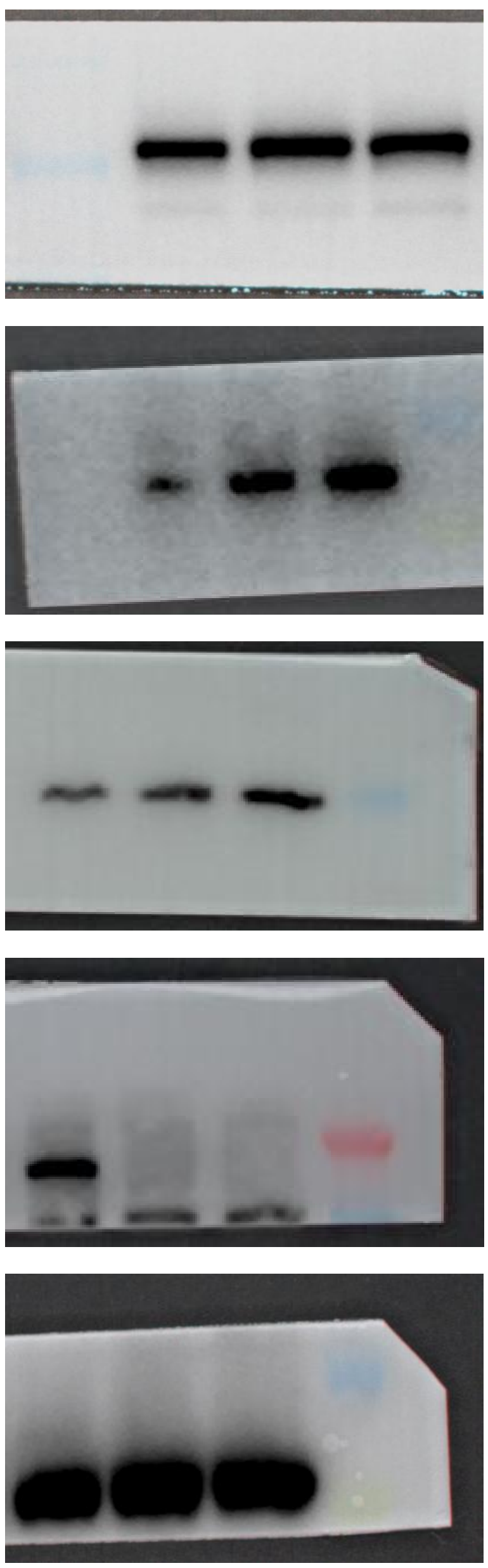

Figure 3G:

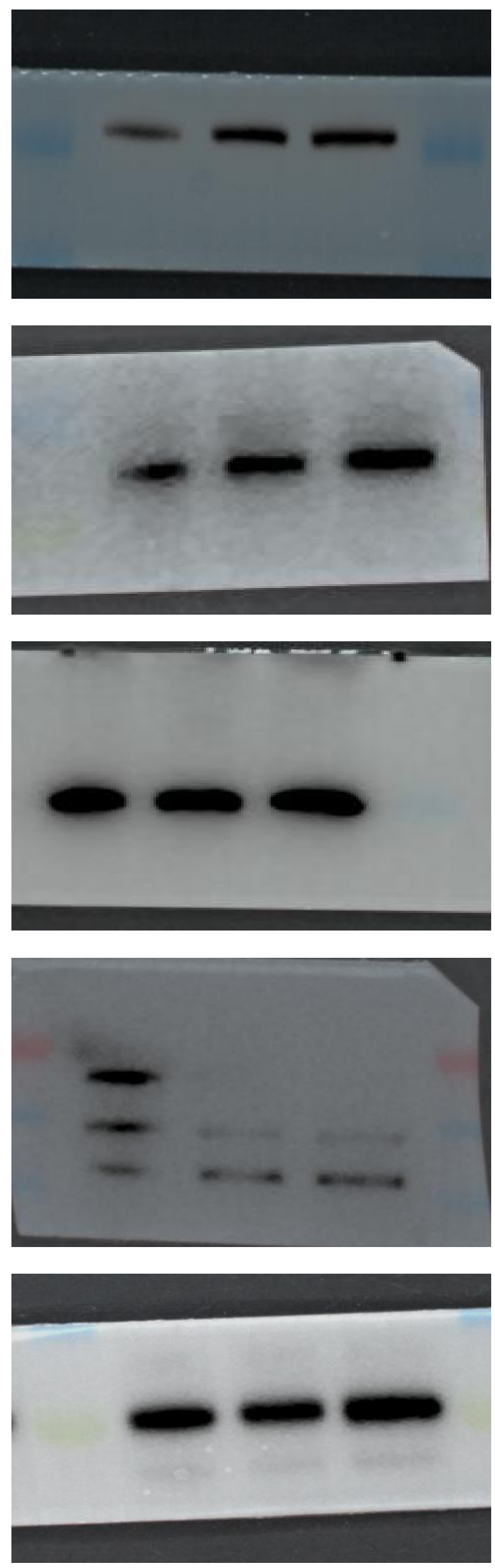

Figure 4F:

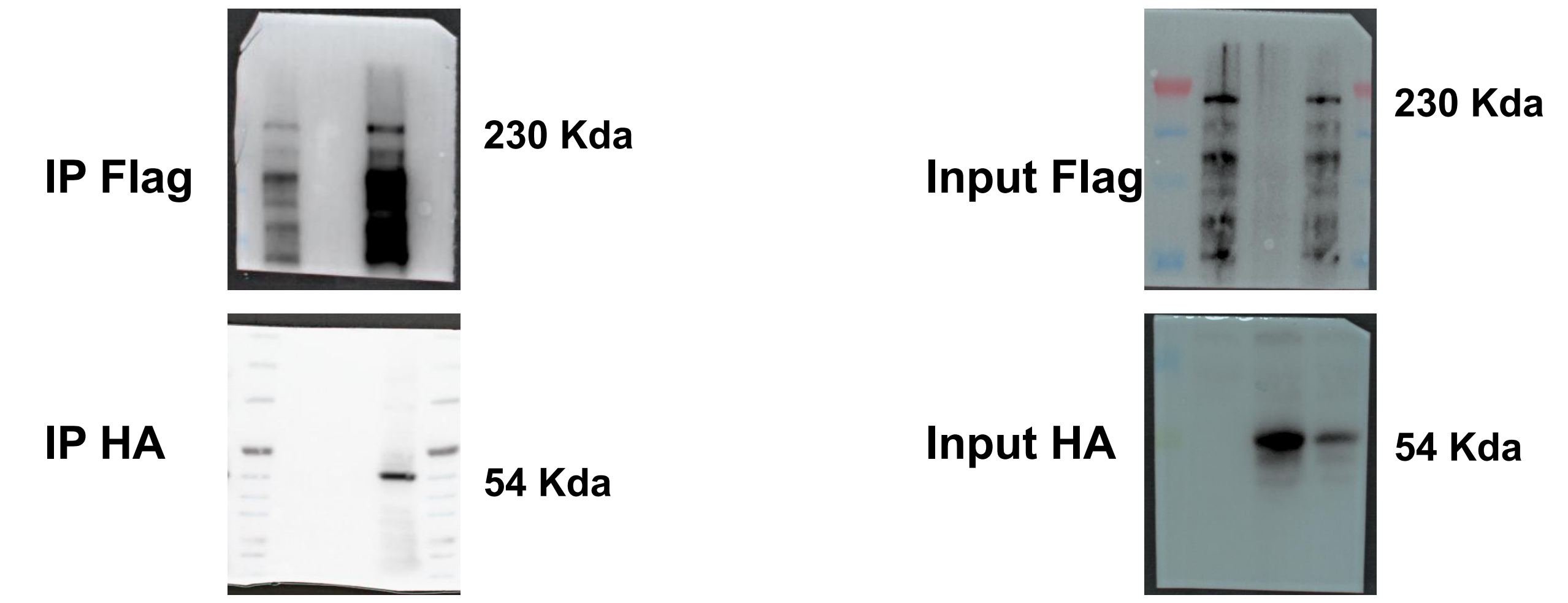

Figure 4G:

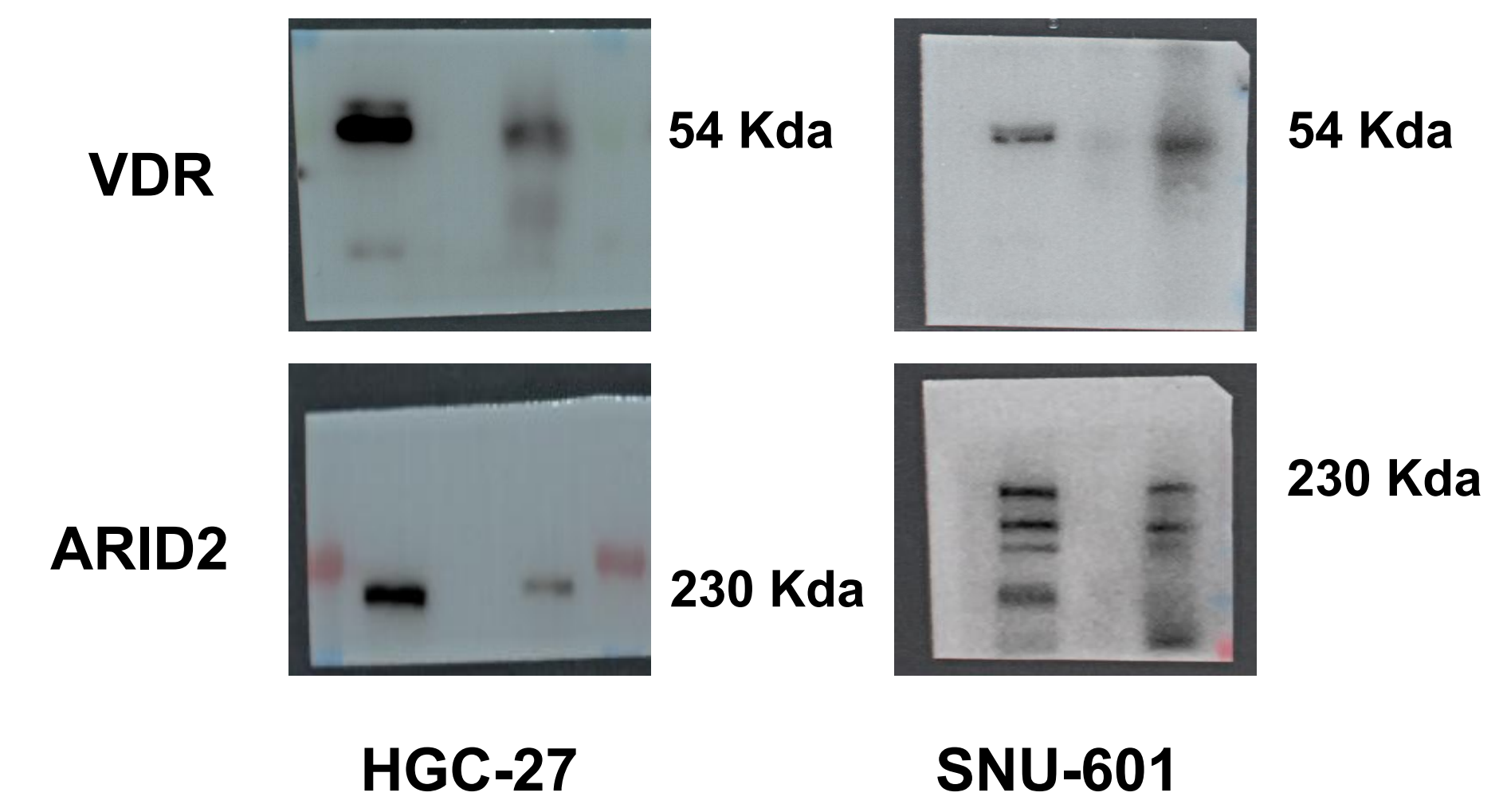

Figure 4H:

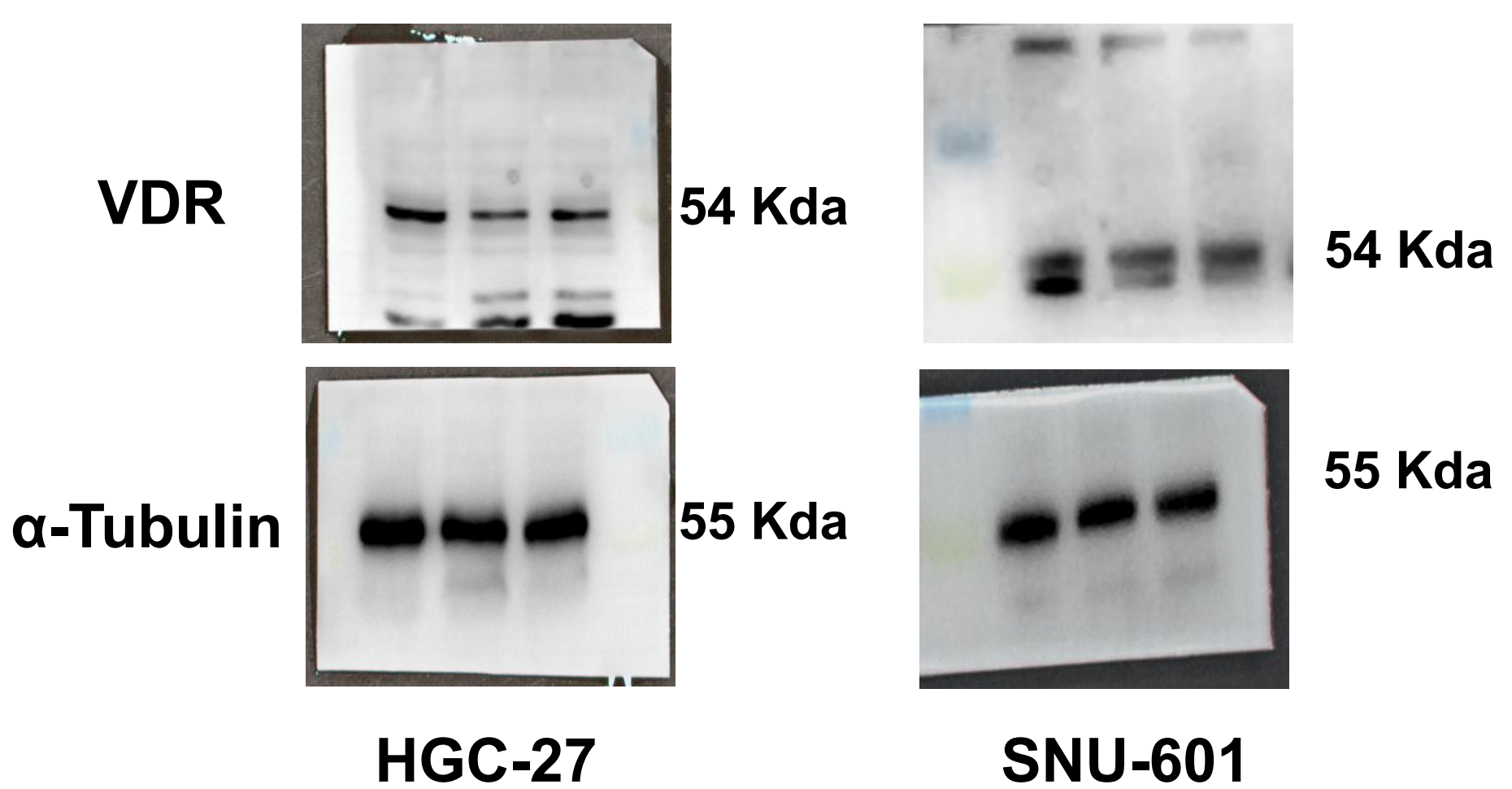

Figure 4I:

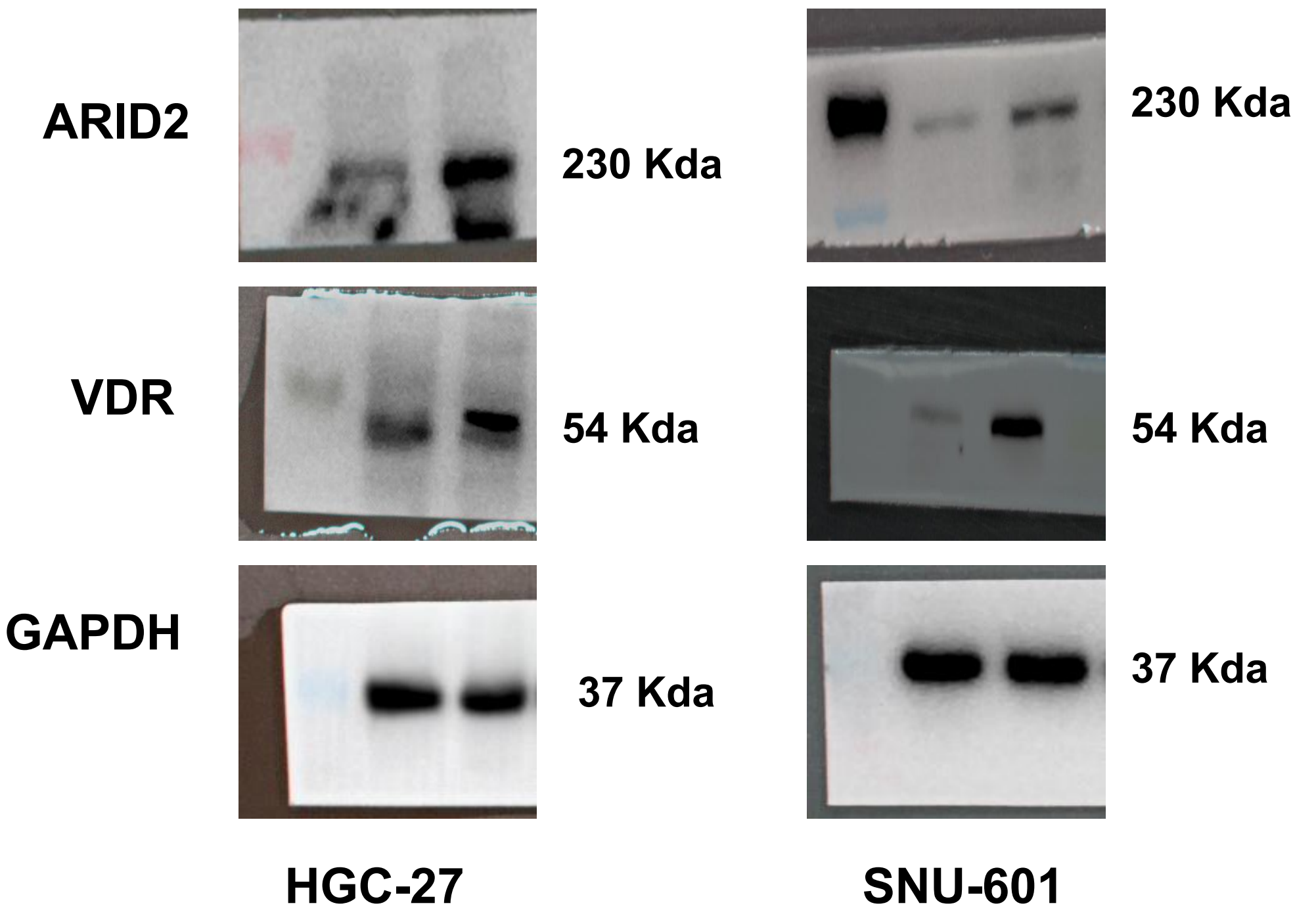

Figure 5C:

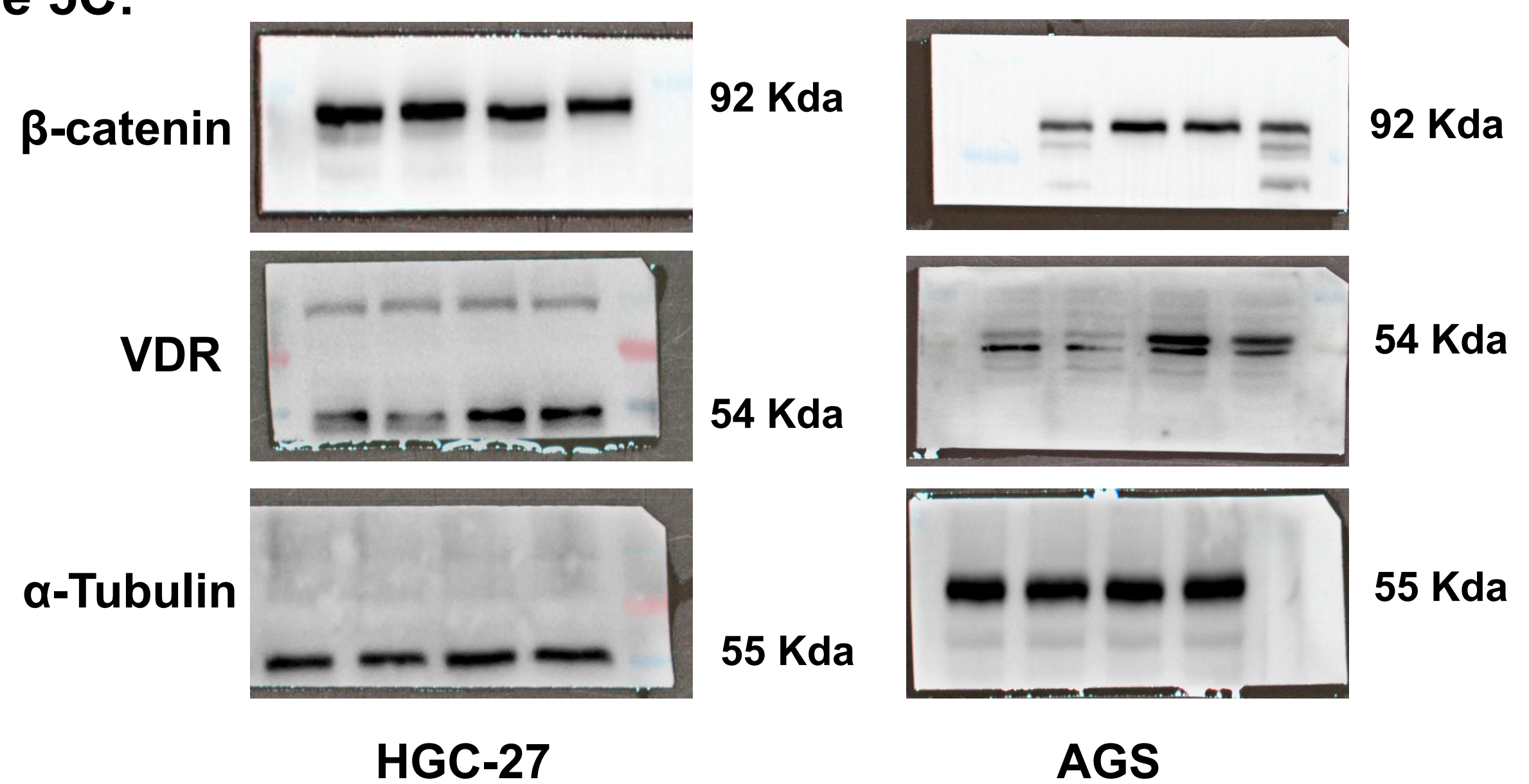

Figure 5G:

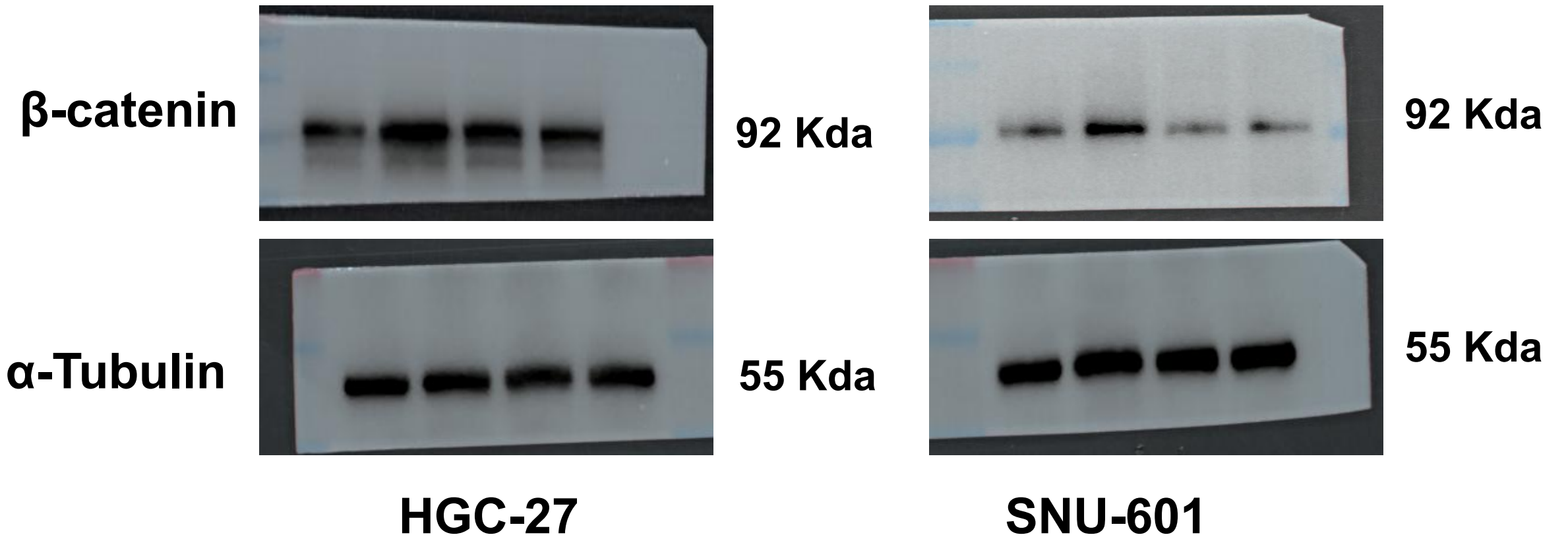

Figure S1:

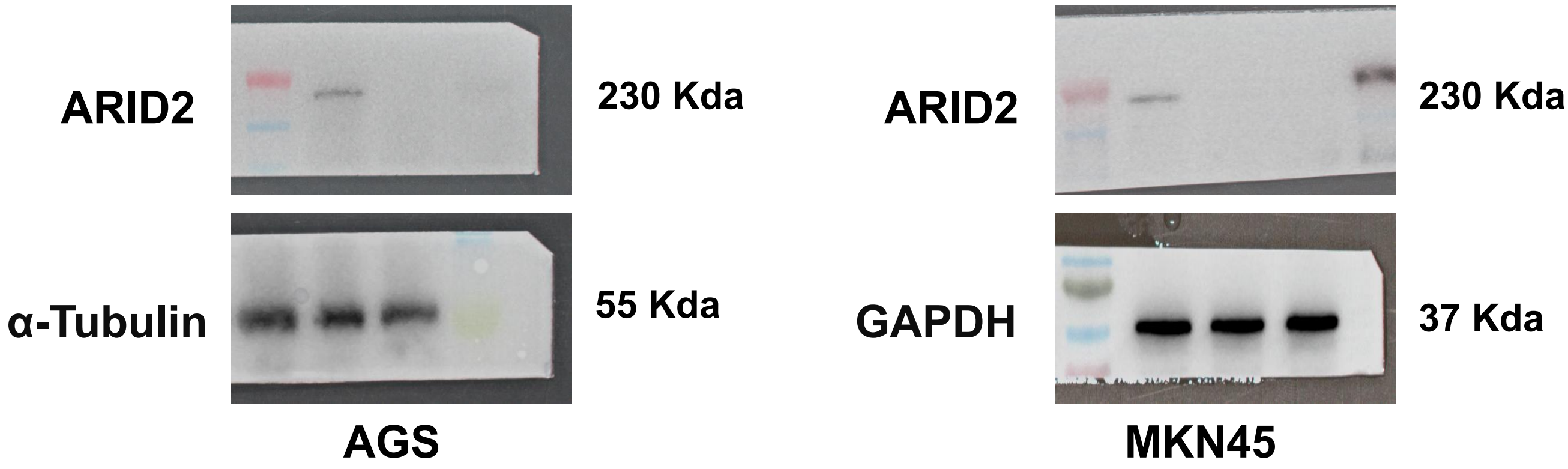

Figure S2D:

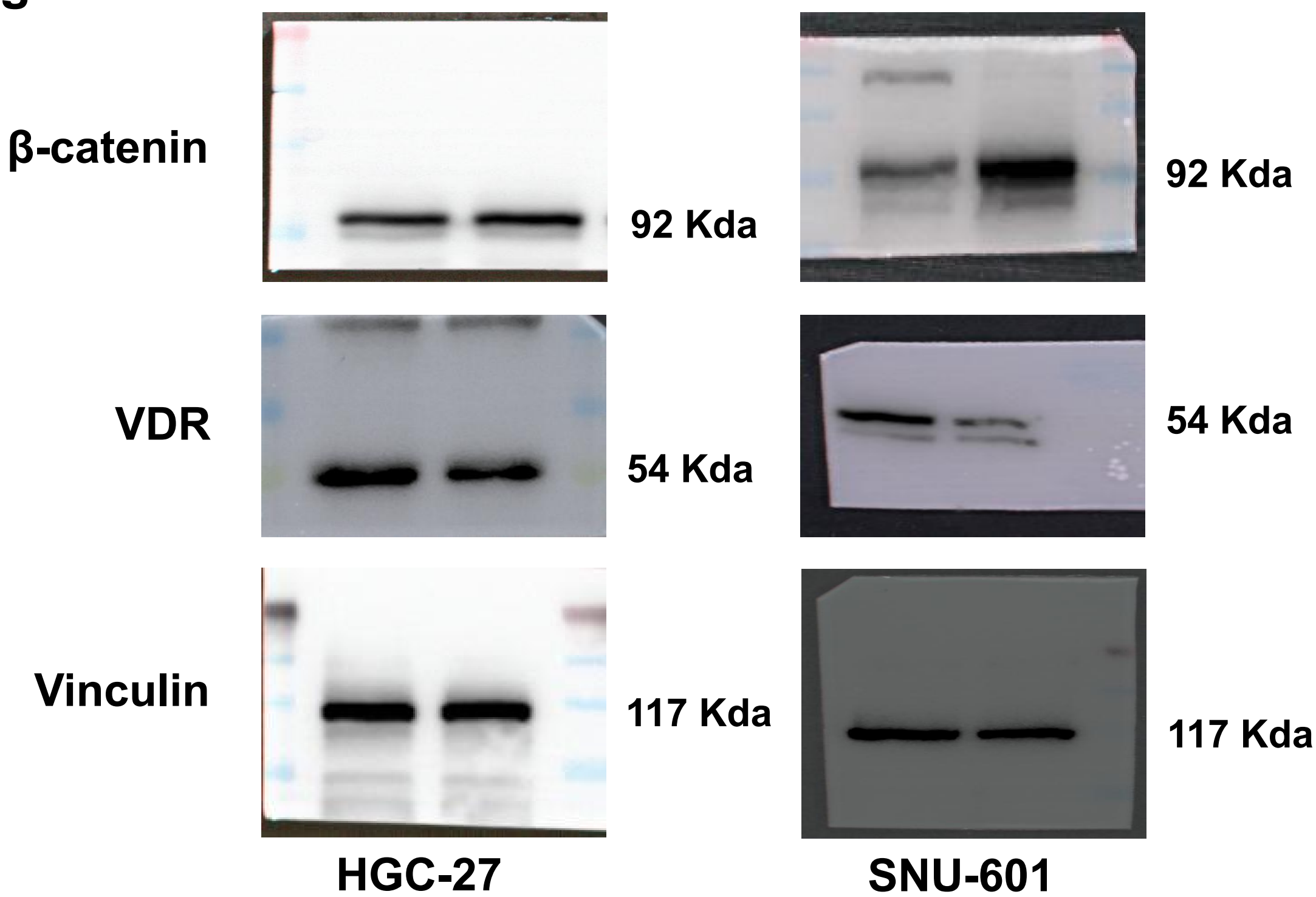

Figure S2E:

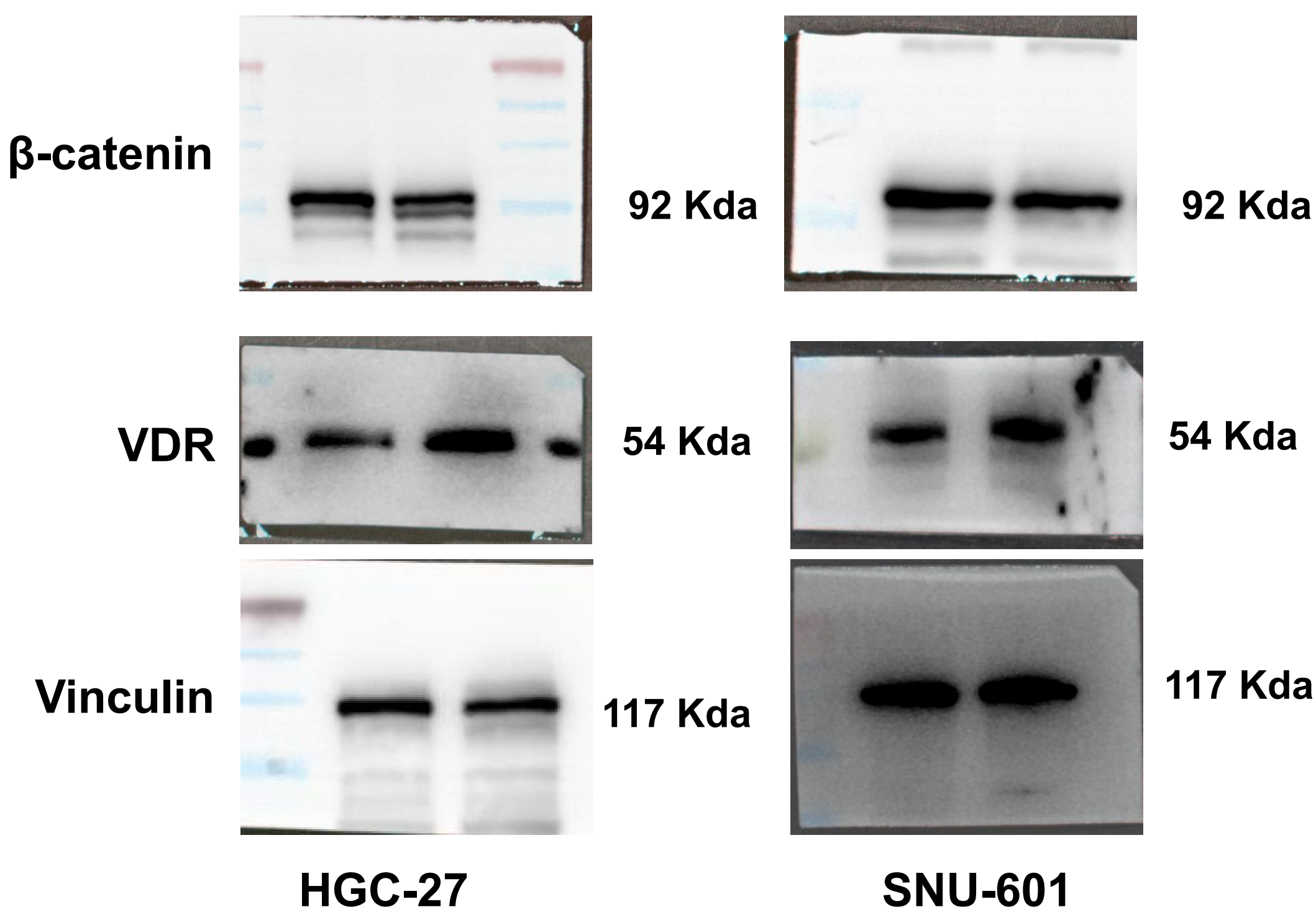

Supplement: Supplementary file 1 [file mmc2.pdf]
