# Supplementary material for: Calcitriol alleviates ARID2-deficient gastric cancer metastasis via inhibition of the Wnt/β-catenin signaling pathway
Source: Transl Oncol. 2026 Sep 22;73:103048. doi: 10.1016/j.tranon.2026.103048 (PMC13633874; doi:10.1016/j.tranon.2026.103048)
Supplement: Supplementary file 2 [file mmc1.docx]

**Supplementary data**

**Calcitriol alleviates ARID2-deficient gastric cancer metastasis via inhibition of the Wnt/β-catenin signaling pathway**

**Supplementary Figures**

**Fig. S1. Analysis of ARID2 expression across different clinical stages in GC.** Data were obtained from the GSE84437 dataset. (A) Box plots showing ARID2 expression across different lymph node metastasis stages (N stage: N0-N3). (B) Box plots showing ARID2 expression across different tumor invasion depths (T stage: T1-T4).

**Fig. S2. Analysis of the impact of ARID2 on cell-matrix adhesion, migration, invasion, and gene regulation.** (A-D) Western blot and RT‒qPCR were used to detect the protein and mRNA levels of ARID2, respectively, in shARID2 GC cells (*n* = 3). (E, F) Representative images and quantification of a cell-matrix adhesion assay using SNU-601 cells transduced with shARID2-1 or shNC (scale bar = 100 μm, *n* = 3). (G, H) Representative images and quantification of the transwell assay using MKN45 cells transduced with shARID2-1 or shNC (scale bar = 200 μm, *n* = 3). (I, J) Representative images and quantification of the wound healing assay using AGS cells transduced with shARID2-1 or shNC (scale bar = 200 μm, *n* = 3). (K) GO analysis for upregulated genes after ARID2 knockdown. (L) Binding peak distribution and proportion in the CUT&Tag assay by anti-ARID2. ns, not significant; * *P* < 0.05, ** *P* < 0.01, *** *P* < 0.001, **** *P* < 0.0001.

**Fig. S3. ARID2 knockdown mediates the transcriptional activation of Wnt signaling by regulating VDR.** (A) Heatmap representing the mRNA expression of VDR target genes in HGC-27 cells with ARID2 knockdown (*n* = 3). (B, C) The mRNA expression of Wnt target genes was analyzed by RT‒qPCR following VDR knockdown or overexpression in GC cells (*n* = 3). (D, E) Western blotting was used to detect the protein levels of β-catenin in shVDR or OE-VDR GC cells (*n* = 3). ns, not significant; * *P* < 0.05, ** *P* < 0.01, *** *P* < 0.001, **** *P* < 0.0001.

**Fig. S4. Low VDR expression is associated with poor prognosis in GC.** (A) Forest plot illustrating the subgroup analysis of hazard ratios for overall survival based on VDR expression in the ACRG cohort. (B) Kaplan‒Meier curves for overall survival in GC patients from the ACRG cohort, stratified by high and low VDR expression. (C) Box plots showing VDR expression levels across different clinical stages in the ACRG cohort. (D) Comparison of VDR expression across distinct molecular subtypes in the ACRG cohort. (E) Stacked bar charts showing the proportion of patients with high or low VDR expression in the metastasis-negative and metastasis-positive groups from the GSE2685 dataset.

**Fig. S5. Treatment with calcitriol activates VDR signaling.** (A, B) The mRNA expression of the VDR signaling-related gene *CYP27B1* was analyzed in GC cells expressing shNC or shVDR (*n* = 3). (C, D) The mRNA expression of the VDR signaling-related gene *CYP27B1* was analyzed in GC cells expressing shVDR or shNC following calcitriol treatment (*n* = 3). ns, not significant; * *P* < 0.05, ** *P* < 0.01, *** *P* < 0.001, **** *P* < 0.0001.

**Fig. S6. VDR overexpression inhibits tumor progression induced by ARID2 knockdown in GC.** (A, B) Cell-matrix adhesion assays of ARID2-knockdown-VDR-overexpressing GC cells (scale bar = 200 μm, *n* = 3). (C, D) Cell-matrix adhesion assays of ARID2-knockdown-calcitriol-treated GC cells (scale bar = 200 μm, *n* = 3). (E, F) The body weights of the mice showed no significant difference among the indicated groups (*n* = 5). * *P* < 0.05, ** *P* < 0.01, *** *P* < 0.001, **** *P* < 0.0001.

**Fig. S7. VDR IHC scores in GC tissues.** (A) Representative IHC staining of VDR in tumor and adjacent normal samples (scale bar = 200 μm, *n* = 80). Para: paracancerous tissues. (B) IHC scores of VDR in GC tumor tissues and adjacent normal tissues (*n* = 73). (C) The distribution of VDR in the low and high ARID2 expression groups. (D) IHC scores of VDR in tumor tissues with different lymph node metastasis stages (N0-1, *n* = 30; N2-3, *n* = 40). * *P* < 0.05, ** *P* < 0.01, *** *P* < 0.001, **** *P* < 0.0001.

**Supplementary Tables**

**Table S1. Primer sequences.**

| Symbol |  | Primer sequence (5’-3’) |
| --- | --- | --- |
| *c-MYC* | F | CCTGGTGCTCCATGAGGAGAC |
|  | R | CAGACTCTGACCTTTTGCCAGG |
| *BMP4* | F | CTGGTCTTGAGTATCCTGAGCG |
|  | R | TCACCTCGTTCTCAGGGATGCT |
| *WNT5A* | F | TACGAGAGTGCTCGCATCCTCA |
|  | R | TGTCTTCAGGCTACATGAGCCG |
| *WNT6* | F | GTGCAACTGCACAACAACGAGG |
|  | R | GAAATGGAGGCAGCTTCTGCCA |
| *CYP24A1* | F | GCTTCTCCAGAAGAATGCAGGG |
|  | R | CAGACCTTGGTGTTGAGGCTCT |
| *VDR* | F | CGCATCATTGCCATACTGCTGG |
|  | R | CCACCATCATTCACACGAACTGG |
| *CYP27B1* | F | CTCCACTCAGAGATCACAGCTG |
|  | R | GGACACGAGAATTTCCAGGTACC |
| *CYP27A1* | F | GTGCTGCCTTTCTGGAAGCGAT |
|  | R | TAGCCAGACACCTGGATGCCAT |
| *CYP2R1* | F | GAAACCTCTGAAGCAATGCTGTAA |
|  | R | TCCTGAGCGATTTCTGGACAGC |
| *CAMP* | F | GACACAGCAGTCACCAGAGGAT |
|  | R | TCACAACTGATGTCAAAGGAGCC |
| *ARID2* | F | CCTGATGCACTAGCTGCGGTAA |
|  | R | GGAGCAACATGCTGCGCTACAA |
| *CCND1* | F | TCTACACCGACAACTCCATCCG |
|  | R | TCTGGCATTTTGGAGAGGAAGTG |
| *GAPDH* | F | GTCTCCTCTGACTTCAACAGCG |
|  | R | ACCACCCTGTTGCTGTAGCCAA |
| *VDR* promoter  primer #1 | F | TGGACGACGGTCGATGAAAA |
|  | R | ACAGGTCCAGTCCTCTCTTG |
| *VDR* promoter  primer #2 | F | AAGTCAAGATGGTTGCAGCG |
|  | R | CGCCTTTTGACAAGCAGAGAC |
| shVDR |  | CCGGCTCCTGCCTACTCACGATAAATTCAAGAGATTTATCGTGAGTAGGCAGGAGTTTTTT |
| shARID2-1 |  | CCGGGTTCTGTTGTTCAGAATCATACTCGAGTATGATTCTGAACAACAGAACTTTTTT |
| shARID2-2 |  | CCGGGCAGGATAAGCACTGTTCAAACTCGAGTTTGAACAGTGCTTATCCTGCTTTTTT |

**Table S2. Antibodies used in this study.**

| **Antibody** | **Type** | **Vendor** | **Catalog No.** | **IDENTIFIER** |
| --- | --- | --- | --- | --- |
| ARID2 | Mouse mAb | Santa | sc-166117 | RRID:AB_2060382 |
| VDR | Rabbit mAb | ABclonal | A23289 | RRID:AB_3720104 |
| β-catenin | Rabbit pAb | Proteintech | 51067-2-AP | RRID:AB_2086128 |
| Cyclin D1 | Rabbit mAb | Proteintech | 60186-1-IG | RRID:AB_10793718 |
| c-Myc | Rabbit mAb | CST | 68309 #5605 | RRID:AB_1903938 |
| DYKDDDDK tag | Mouse mAb | Proteintech | 66008-4-IG | RRID:AB_2918475 |
| HA tag | Rabbit pAb | Proteintech | 51064-2-AP | RRID:AB_11042321 |
| Vinculin | Rabbit pAb | Proteintech | 66305-1-IG | RRID:AB_2810300 |
| GAPDH | Mouse mAb | ABclonal | AC002 | RRID:AB_2736879 |
| Alpha Tubulin | Rabbit pAb | Proteintech | 11224-1-AP | RRID:AB_2210206 |
